# Configuration balancing for stochastic requests

**DOI:** 10.1007/s10107-024-02132-w

**Published:** 2024-08-08

**Authors:** Franziska Eberle, Anupam Gupta, Nicole Megow, Benjamin Moseley, Rudy Zhou

**Affiliations:** 1 Department of Mathematics, Technische Universität Berlin, Straße des 17. Juni 136, 10623 Berlin, Germany; 2 Computer Science Department, New York University, 251 Mercer Street, New York, NY 10012 USA; 3 Faculty of Mathematics and Computer Science, University of Bremen, Bibliotheksstr. 5, 28359 Bremen, Germany; 4 Tepper School of Business, Carnegie Mellon University, 5000 Forbes Ave, Pittsburgh, PA 15213 USA

**Keywords:** Stochastic scheduling, Stochastic routing, Load balancing, Unrelated machines

## Abstract

The configuration balancing problem with stochastic requests generalizes well-studied resource allocation problems such as load balancing and virtual circuit routing. There are given *m* resources and *n* requests; each request has multiple possible *configurations*, each of which increases the load of each resource by some amount. The goal is to select one configuration for each request to minimize the *makespan*: the load of the most-loaded resource. In the stochastic setting, the amount by which a configuration increases the resource load is uncertain until the configuration is chosen, but we are given a probability distribution. We develop both offline and online algorithms for configuration balancing with stochastic requests. When the requests are known offline, we give a non-adaptive policy for configuration balancing with stochastic requests that $O(\frac{\log m}{\log \log m})$-approximates the optimal adaptive policy, which matches a known lower bound for the special case of load balancing on identical machines. When requests arrive online in a list, we give a non-adaptive policy that is $O(\log m)$ competitive. Again, this result is asymptotically tight due to information-theoretic lower bounds for special cases (e.g., for load balancing on unrelated machines). Finally, we show how to leverage adaptivity in the special case of load balancing on *related* machines to obtain a constant-factor approximation offline and an $O(\log \log m)$-approximation online. A crucial technical ingredient in all of our results is a new structural characterization of the optimal adaptive policy that allows us to limit the correlations between its decisions.

## Introduction

This paper considers the *configuration balancing* problem: there are *m* resources and *n* requests. Request *j* has $q_j$ configurations $x_j(1), \ldots , x_j(q_j) \in \mathbb {R}_{\ge 0}^m$. We must choose one configuration $c_j \in [q_j]$ per request, which adds $x_j(c_j)$ to the load vector on the resources. The goal is to minimize the makespan, i.e., the load of the most-loaded resource. Configuration balancing captures many natural resource allocation problems where requests compete for a finite pool of resources and the task is to find a “fair” allocation in which no resource is over-burdened. Two well-studied problems of this form arise in scheduling and routing.

(i) In *load balancing* a.k.a. *makespan minimization*, there are *m* (unrelated) machines and *n* jobs. Scheduling job *j* on machine *i* increases the load of *i* by $p_{ij} \ge 0$. The goal is to schedule each job on some machine to minimize the makespan, i.e., the load of the most-loaded machine.
(ii) In *virtual circuit routing* or *congestion minimization*, there is a directed graph $G=(V,E)$ on *m* edges with edge capacities $s_e > 0$ for $e \in E$, and *n* requests, each request consisting of a source-sink pair $(a_j, b_j)$ in *G* and a demand $x_j\ge 0$. The goal is to route each request *j* from $a_j$ to $b_j$ via some directed path, increasing the load/congestion of each edge *e* on the path by , while the objective is to minimize the load of the most-loaded edge.

Configuration balancing captures both problems by taking the *m* resources to be the *m* machines or edges, respectively; each configuration now corresponds to assigning a job to some machine or routing a request along some path.

Typically, job sizes or request demands are not known exactly when solving resource allocation problems in practice. This motivates the study of algorithms under uncertainty, where an algorithm must make decisions given only partial/uncertain information about the input. Uncertainty can be modeled in different ways. In exceptional cases, a *non-clairvoyant* algorithm that has *no* knowledge about the loads of requests may perform surprisingly well; an example is Graham’s greedy list scheduling for load balancing on identical machines [2]. In general, a non-clairvoyant algorithm cannot perform well. Hence, we consider a stochastic model, where the unknown input follows some known distribution but the actual realization is a priori unknown. Such a model is natural when there is historical data available from which such distributions can be deduced.

In the *configuration balancing with stochastic requests* problem, we assume that each configuration *c* of request *j* is a random vector $X_j(c)$ with known distribution $\mathcal {D}_{j}(c)$ supported on $\mathbb {R}_{\ge 0}^m$ such that the $X_j(c)$’s are independent across different requests *j*. The actual realized vector of a configuration *c* of request *j* is only observed after *irrevocably* selecting this particular configuration for request *j*. The objective is to minimize the expected maximum load (makespan)

$$ \mathbb {E} \Big [\max _i \sum _{j=1}^n X_{ij}(c_j) \Big ], $$

where $c_j$ is the configuration chosen for request *j*. We assume that we have oracle access to the $\mathcal {D}_{j}(c)$’s; in particular we assume that in constant time, we can compute any needed statistic of the distribution $\mathcal {D}_j(c)$. Thus, configuration balancing with stochastic requests captures natural stochastic generalizations of the above two problems:

(i) In *load balancing with stochastic jobs*, there are *m* (unrelated) machines and *n* jobs. Scheduling job *j* on machine *i* increases the load of *i* by a random size $X_{ij} \ge 0$ with known distribution such that the $X_{ij}$’s are independent across *j*’s. The goal is to adaptively schedule the jobs (upon scheduling job *j* to machine *i*, we observe the realized value of $X_{ij}$) to minimize the expected makespan.
(ii) In *virtual circuit routing with stochastic requests*, there is a directed graph $G=(V,E)$ on *m* edges with edge capacities $s_e > 0$ for $e \in E$, and *n* requests, each request consisting of a source-sink pair $(a_j, b_j)$ in *G* and a random demand $X_j \ge 0$ with known distribution such that the $X_j$’s are independent across *j*’s. The goal is to adaptively choose a $a_j$ - $b_j$ path for each request *j* such that upon choosing such a path, we observe the realized value of $X_j$ and the load of each edge *e* on the path increases by . The objective is to minimize the expected load of the most-loaded edge.

Further, we distinguish whether there is an additional dimension of uncertainty or not, namely the knowledge about the request set. In the *offline* setting, the set of requests and the distributions of the configurations of each request are known up-front, and they can be selected and assigned to the resources irrevocably in any order. In the *online* setting, requests are not known in advance and they are revealed one-by-one (online-list model). The algorithm learns the stochastic information on configurations of a request upon its arrival, and must select one of them without knowledge of future arrivals. After a configuration is chosen irrevocably, the next request arrives.

In general, we allow an algorithm to base the next decision on knowledge about the realized vectors of all previously selected request configurations. That is, a policy can select a configuration, say *c*, of request *j*, observe the realized values of the vector $X_j(c)$, and can make subsequent decisions based on this information. We call such policies *adaptive*. Conversely, a *non-adaptive* policy is one that fixes the particular configuration chosen for a request without using any knowledge of the realized configuration vectors. We define the *adaptivity gap* for configuration balancing for stochastic requests as $\max _{\mathcal {I}} \frac{\mathbb {E}[\textsc {Opt}_{N\!A}(\mathcal {I})]}{\mathbb {E}[\textsc {Opt}_{A}(\mathcal {I})]}$, where the maximum is taken over all instances $\mathcal {I}$ and $\textsc {Opt}_{N\!A}$ and $\textsc {Opt}_{A}$ are the makespan of the optimal non-adaptive and adaptive policies, respectively.

The goal of this paper is to investigate the power of adaptive and non-adaptive policies for online and offline configuration balancing with stochastic requests. We quantify the performance of an algorithm Alg  by comparing its achieved expected makespan $\mathbb {E}[\textsc {Alg}(\mathcal I)]$ on instance $\mathcal I$ with $\mathbb {E}[\textsc {Opt}(\mathcal I)]$, the expected makespan of an optimal offline adaptive policy Opt  that can process the requests in any order: More precisely, we bound the worst case ratio $\max _{\mathcal {I}} \frac{\mathbb {E}[\textsc {Alg}(\mathcal {I})]}{\mathbb {E}[\textsc {Opt}(\mathcal {I})]}$, where again the maximum is taken over all instances $\mathcal {I}$. We say $\textsc {Alg}$ is an $\alpha$-*approximation* if its approximation ratio is at most $\alpha$. Similarly, for an *online* algorithm $\textsc {Alg}$, we say it is $\alpha$-*competitive* if its approximation ratio is at most $\alpha$.

One subtlety in our stochastic model is that an online algorithm must process the requests in a fixed order, while we allow the offline optimum to adaptively choose an order to process the requests and thus reveal different information about their realized values. Another possible model that has been considered in other works [3] is to compare to the offline optimum that processes the requests in the same fixed order (known offline). In the deterministic setting, the offline adaptive and fixed order optima coincide, but this is not the case in the stochastic setting. Thus, upper bounds on the competitive ratio for our model translate to upper bounds on the gap between adaptive- and fixed-order policies.

### Our results

As our first main result, we present non-adaptive algorithms for offline and online configuration balancing with stochastic requests.

#### Theorem 1

For configuration balancing with stochastic requests there is a randomized *offline* algorithm that computes a non-adaptive policy that is a $\Theta \big (\frac{\log m}{\log \log m}\big )$-approximation and an efficient deterministic *online* algorithm that is a $\Theta (\log m)$-approximation when comparing to the optimal offline adaptive policy. Both algorithms run in polynomial time if the configuration distributions are given explicitly as input.

In the above theorem, we assume that each configuration distribution has finite support and is specified by the value-probability pairs for each atom of the distribution. See Lemma 10 for a more general statement that is agnostic to the encoding of the distributions.

The offline analysis relies on a linear programming (LP) relaxation of configuration balancing, which has a known integrality gap of $\Theta \big (\frac{\log m}{\log \log m}\big )$, even for virtual circuit routing [4], implying that the analysis is tight—see Appendix C for formal definition of integrality gap and proof sketch. In the online setting, our analysis employs a potential function to greedily determine which configuration to choose for each request. In particular, we generalize the idea by [5] to the setting of configuration balancing with stochastic requests and match a known lower bound for online deterministic load balancing on unrelated machines by [6].

If the configurations are not given explicitly as part of the input or the number of configurations is large, then efficiently solving the problem requires us to be able to optimize over configurations in polynomial time.

***Applications:***

These results would hold for both load balancing on unrelated machines and virtual circuit routing if we could guarantee that either the configurations are given explicitly or the respective subproblems can be solved efficiently. We can ensure this in both cases.

For stochastic load balancing on unrelated machines, the resources are the *m* machines, and each job has *m* possible configurations—one corresponding to assigning that job to each machine. Thus, we can efficiently represent all configurations. Further, here the LP relaxation of configuration balancing used in Theorem 1 is equivalent to the LP relaxation of the generalized assignment problem (GAP) solved in [7], which gives a deterministic rounding algorithm. Hence, Theorem 1 implies the following theorem.

#### Theorem 2

There exist efficient deterministic algorithms that compute a non-adaptive policy for load balancing on unrelated machines with stochastic jobs that achieve an $\Theta \big (\frac{\log m}{\log \log m}\big )$-approximation *offline* and an $\Theta (\log m)$-approximation *online* when comparing to the optimal offline adaptive policy.

These results are asymptotically tight due to the lower bound of $\Omega \big (\frac{\log m}{\log \log m}\big )$ on the adaptivity gap [8] and the lower bound of $\Omega (\log m)$ on the competitive ratio of any deterministic online algorithm, even for deterministic requests [6]. This implies that the adaptivity gap for stochastic load balancing is $\Theta \big (\frac{\log m}{\log \log m}\big )$.

For virtual circuit routing, the resources are the *m* edges and each request has a configuration for each possible routing path. Thus, efficiently solving the subproblems requires more work as the configurations are only given *implicitly* and there can be exponentially many. For the offline setting, since the LP relaxation has (possibly) exponentially many variables, we design an efficient separation oracle for the dual LP in order to efficiently solve the primal. For the online setting, we carefully select a subset of polynomially many configurations that contain the configuration chosen by the greedy algorithm, even when presented with all configurations. Thus, Theorem 1 implies that stochastic requests are not harder to approximate than deterministic requests.

#### Theorem 3

For routing with stochastic requests, there exist an efficient randomized *offline* algorithm computing a non-adaptive policy that is a $\Theta \big (\frac{\log m}{\log \log m}\big )$-approximation and an efficient deterministic *online* algorithm that computes an $\Theta (\log m)$-approximation when comparing to the optimal offline adaptive policy.

#### Adaptive policies for related machines

When each request *j* has *m* configurations and configuration $c \in [m]$ can be written as $X_{j}(c) = \frac{X_j}{s_i} \, e_{c}$, where $e_c \in \mathbb {R}^m$ is the *c*th standard unit vector, the problem is also known as *load balancing on related machines*. We say that $X_j$ is the size of request (or job) *j* and $s_i$ is the speed of resource (or machine) *i*. In this special case, we show how to leverage adaptivity to overcome the $\Omega \big (\frac{\log m}{\log \log m}\big )$ lower bound on the adaptivity gap. Interestingly, our adaptive algorithms begin with a similar *non-adaptive* assignment of jobs to machines, but we deviate from the assignment adaptively to obtain our improved algorithms.

##### Theorem 4

For load balancing on related machines with stochastic jobs, there exist efficient algorithms that compute an adaptive *offline* 432-approximation and an adaptive *online* $O(\log \log m)$-approximation when comparing to the optimal offline adaptive policy.

We note that we have not tried to optimize the constant for our offline algorithm. It remains an interesting open question whether the online setting admits an *O*(1)-competitive algorithm.

As adaptivity turns out to be very powerful in load balancing on related machines, it is reasonable to ask whether the (stochastic) information about job sizes is even needed to improve upon Theorem 1. We answer this question to the affirmative in the following sense. In Appendix B, we show that *non-clairvoyant algorithms*, that have no prior knowledge of the job sizes, approximate the optimal offline schedule only within a factor $\Omega (\sqrt{m})$, even if the size of a job is revealed immediately upon assigning it to a machine. Further, we give a non-clairvoyant algorithm that is $O(\sqrt{m})$-approximate, asymptotically matching this lower bound.

### Technical overview

We illustrate the main idea behind our non-adaptive policies, which compare to the optimal offline adaptive policy. Throughout this paper, we let $\textsc {Opt}$ denote the optimal adaptive policy as well as its makespan. As in many other stochastic optimization problems, our goal is to give a good deterministic proxy for the makespan of a policy. Then, our algorithm will optimize over this deterministic proxy to obtain a good solution. First, we observe that if all configurations were bounded with respect to $\mathbb {E}[\textsc {Opt}]$ in every entry, then selecting configurations such that each resource has expected load $O(\mathbb {E}[\textsc {Opt}])$ gives the desired $O\big (\frac{\log m}{\log \log m}\big )$-approximation by standard concentration inequalities for independent sums with bounded increments. Thus, in this case the expected load on each resource is a good proxy. However, in general, we have no upper bound on $X_{ij}(c)$, so we cannot argue as above. We turn these unbounded random variables (RVs) into bounded ones in a standard way by splitting each request into *truncated* and *exceptional* parts.

#### Definition 5

(*Truncated and Exceptional Parts*) Fix $\tau \ge 0$ as threshold. For a RV *X*, its truncated part (w.r.t. threshold $\tau$) is $X^T:= X \cdot \mathbbm {1}_{X < \tau }$ and its exceptional part is $X^E:= X \cdot \mathbbm {1}_{X \ge \tau }$. Note that $X = X^T + X^E$.

It is immediate that the truncated parts $X_{ij}^T(c)$ are bounded in $[0, \tau ]$. Taking $\tau = O(\mathbb {E}[\textsc {Opt}])$, we can control their contribution to the makespan using concentration. It remains to find a good proxy for the contribution of exceptional parts to the makespan. This is one of the main technical challenges of our work as we aim to compare against the optimal adaptive policy: adaptive policies have much better control over the exceptional parts than non-adaptive ones.

Concretely, let $c_j$ be the configuration chosen by some fixed policy for request *j*. Note that $c_j$ itself can be a random variable in $\{1,\ldots ,q_j\}$. We want to control the quantity

$$ \mathbb {E}\big [\max _i \sum _{j=1}^n X_{ij}^E(c_j) \big ]. $$

Since we have no reasonable bound on the $X_{ij}^E(c_j)$’s, for non-adaptive policies, we can only upper bound the expected maximum by the following sum

1 $$\begin{aligned} \mathbb {E}\Big [\max _{1 \le i \le m} \sum _{j=1}^n X_{ij}^E(c_j) \Big ] \le \sum _{j=1}^n \mathbb {E}\Big [\max _{1 \le i \le m} X_{ij}^E(c_j) \Big ]. \end{aligned}$$

We call the right hand side *total (expected) exceptional load*. The above inequality is tight up to constants for non-adaptive policies, so it seems like the total expected exceptional load is a good proxy to use for our algorithm. However, it is far from tight for adaptive policies as the following example shows.

#### Example 6

Recall that in load balancing on related machines, each request *j* has *m* configurations and configuration $c \in [m]$ has the special form of $X_{j}(c) = \frac{X_j}{s_i} \, e_{c}$, where $X_j$ is the processing time of job *j* and $s_i$ is the speed of machine *i*. We assume that there is one fast machine with speed $s_1 = 1$ and $m-1$ slow machines with speed $s_2 = \cdots = s_m = \frac{1}{\tau m}$, where $\tau > 1$ is the truncation threshold. There are *m* jobs: a stochastic one with processing time $X_j \sim \tau \cdot$Ber$\big (\frac{1}{\tau }\big )$ and $m-1$ deterministic jobs with processing time $X_j \equiv \frac{1}{m}$. The optimal adaptive policy first schedules the stochastic job on the fast machine. If its realized size is 0, then it schedules all deterministic jobs on the fast machine as well. Otherwise the realized size is $\tau$ and it schedules one deterministic job on each slow machine, implying $\mathbb {E}[\textsc {Opt}] = \big (1 - \frac{1}{\tau }\big )\big (\frac{m-1}{m}\big ) + \frac{1}{\tau } \cdot \tau = \Theta (1)$. However, the total expected exceptional load (w.r.t. $\tau$) is $\sum _{i,j} \mathbb {E} \big [X_{ij}^E \cdot \mathbbm {1}_{j \rightarrow i}\big ] = \frac{1}{\tau }(m \tau ) = m$, where $j \rightarrow i$ denotes that job *j* is assigned to machine *i*, i.e., configuration *i* is chosen for *j*.

In the example, the optimal adaptive policy accrues a lot of exceptional load, but this does not have a large effect on the makespan. Concretely, (1) can be loose by a $\Omega (m)$-factor for adaptive policies. Thus, it seems that the total exceptional load is a bad proxy in terms of lower-bounding $\textsc {Opt}$. However, we show that, by comparing our algorithm to a *near-optimal* adaptive policy rather than the optimal one, the total exceptional load becomes a good proxy in the following sense. This is the main technical contribution of our work, and it underlies all of our algorithmic techniques.

#### Theorem 7

For configuration balancing with stochastic requests, there exists an adaptive policy with expected maximum load and total expected exceptional load at most $2 \cdot \mathbb {E} [\textsc {Opt}]$ with respect to any truncation threshold $\tau \ge 2 \cdot \mathbb {E}[\textsc {Opt}]$. Further, any configuration *c* selected by this policy satisfies $\mathbb {E}\big [\max _i X_i(c)\big ] \le \tau$.

The proof of the above relies on carefully modifying the “decision tree” representing the optimal adaptive policy. In light of Theorem 7, the deterministic proxies we consider are the expected truncated load on each resource and the total expected exceptional load. All of our algorithms then proceed by ensuring that both quantities are bounded with respect to $\mathbb {E}[\textsc {Opt}]$. In the offline case, we round a natural assignment-type linear program (LP), and in the online case, we use a potential-function argument. All of these algorithms actually output non-adaptive policies. For the special case of related-machines load balancing, we also compute a non-adaptive assignment but instead of following it exactly, we deviate using adaptivity and give improved solutions.

### Related work

While stochastic optimization problems have long been studied [9, 10], approximation algorithms for them are more recent [11, 12]. By now, multi-stage stochastic problems (where uncertain information is revealed in stages) are well-understood [13–15]. In contrast, more dynamic models, where the exact value of an unknown parameter becomes known at times depending on the algorithms decisions (such as serving a request) still remain poorly understood. Some exceptions come from stochastic knapsack [16–19] as well as stochastic scheduling and routing which we discuss below.

For load balancing with deterministic sizes, a 2-approximation in the most general unrelated-machines offline setting [20] is known. For identical machines ($p_{ij} = p_j$ for all jobs *j*), the greedy algorithm (called *list scheduling*) is a $\big (2 - \frac{1}{m}\big )$-approximation algorithm [2]. This guarantee holds even when the jobs arrive online and *nothing* is known about job sizes. This implies a $\big (2 - \frac{1}{m}\big )$-approximate *adaptive* policy for stochastic load balancing on identical machines.

Apart from this, prior work on stochastic scheduling has focused on approximating the optimal *non-adaptive* policy. There are non-adaptive *O*(1)-approximations known for identical machines [21], unrelated machines [8], the $\ell _q$-norm objective [22], and Top-$\ell$-norms [23].

In contrast, our work focuses on approximating the stronger optimal *adaptive* policy. The *adaptivity gap* (the ratio between the expected makespan of the optimal adaptive and non-adaptive policies) can be $\Omega \big (\frac{\log m}{\log \log m}\big )$ even for the simplest case of identical machines [8]. Thus, previous work on approximating the optimal non-adaptive policy does not immediately give any non-trivial approximation guarantees for our setting. The only previous work on adaptive stochastic policies for load-balancing (beyond the highly-adaptive list scheduling) is by [24]. They propose scheduling policies whose degree of adaptivity can be controlled by parameters and show an approximation factor of $O(\log \log m)$ for scheduling on identical machines.

Other objectives have been studied in stochastic scheduling, such as minimizing the expected sum of (weighted) completion times. Most known adaptive policies have an approximation ratio depending on parameters of the distribution [25–30], with the notable (still polylogarithmic) exception in [31].

Online load balancing with deterministic jobs is also well studied [32]. On identical machines, the aforementioned list scheduling algorithm [2] is $\big (2 - \frac{1}{m}\big )$-competitive. For unrelated machines, there is a deterministic $O(\log m)$-competitive algorithm [5] and this is best possible [6]. When the machines are uniformly related, [33] design an *O*(1)-competitive algorithm for minimizing the makespan. Im et al. [34, 35] study the multi-dimensional generalization to vector scheduling under the makespan and the $\ell _q$-norm objective.

For our particular formulation of configuration balancing, the techniques of [5] give an $O(\log m)$-competitive algorithm for deterministic requests. There are also results for packing integer programs [36–38]. Finally, one can generalize the objective from minimizing the $\ell _\infty$-norm of the machine loads to minimizing more general norms, which is studied under the name *generalized load balancing* in [39].

For oblivious routing with stochastic demands, [40] give an algorithm which is an $O(\log ^2 n)$-approximation with high probability. Here, “oblivious” refers to the requirement that the chosen path between a source-sink pair must not depend on the current congestion of the network. In particular, after specifying a set of paths for each possible source-sink pair, a demand matrix is drawn from an a-priori known distribution and each demand needs to be routed along one of the predefined paths. The obliviousness requirement is very different from our setting and makes the two models essentially incomparable.

When $d_j = 1$ for each source-sink pair, there is an $O\big ( \frac{\log m}{\log \log m}\big )$-approximation algorithm by [41], which is best possible, unless $NP \subseteq ZPTIME (n^{\log \log n})$ [42].

In the online setting, when the source-sink pairs arrive online in a list and have to be routed before the next pair arrives, [5] give a lower bound of $\Omega (\log n)$ on the competitive ratio of any deterministic online algorithm in directed graphs, where *n* is the number of vertices. They also give a matching upper bound. For more details on online routing we refer to the survey [43].

## Configuration balancing with stochastic requests

In this section, we prove our main results for the most general problem we consider: configuration balancing. We give an $O\big (\frac{\log m}{\log \log m}\big )$-approximation offline and an $O(\log m)$-approximation online; both algorithms are non-adaptive. Before describing the algorithms, we give our main structural theorem that enables all of our results. Roughly, we show that instead of comparing to the optimal adaptive policy, by losing only a constant factor in the approximation ratio, we can compare to a near-optimal policy that behaves like a non-adaptive one (w.r.t. the proxy objectives we consider, namely, the total expected exceptional load).

### A structural theorem

In this section, we show that there exists a near-optimal policy as guaranteed by Theorem 7. To this end, we modify the optimal policy by “restarting” whenever an exceptional request is encountered. Additionally, we ensure that this modified policy never selects a configuration *c* for a request *j* with $\mathbb {E}\big [\max _i X_{ij}(c) \big ] > \tau$.

We let *J* denote the set of requests. For any subset $J' \subseteq J$, we let $\textsc {Opt}(J')$ denote the optimal adaptive policy (and its maximum load) on the set of requests $J'$. Note that $\textsc {Opt}(\emptyset ) = 0$. Our (existential) algorithm to construct such a policy will begin by running the optimal policy $\textsc {Opt}(J)$ on all requests. However, once an exceptional request is encountered or the next decision will choose a configuration with too large expected maximum, we cancel $\textsc {Opt}(J)$ and instead recurse on all remaining requests, ignoring all previously-accrued loads; see Algorithm 1. The idea of our analysis is that we recurse with small probability.

**Algorithm 1:**
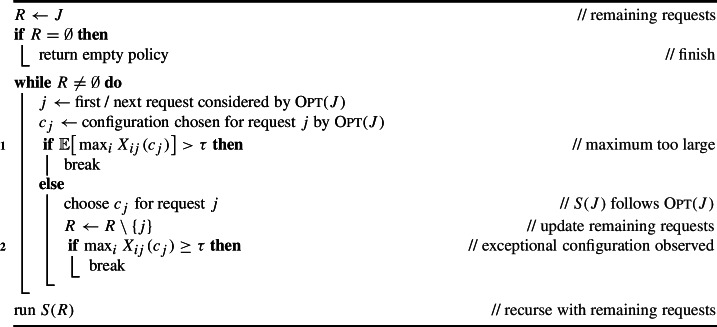
Policy *S*(*J*)

#### Theorem 7

For configuration balancing with stochastic requests, there exists an adaptive policy with expected maximum load and total expected exceptional load at most $2 \cdot \mathbb {E} [\textsc {Opt}]$ with respect to any truncation threshold $\tau \ge 2 \cdot \mathbb {E}[\textsc {Opt}]$. Further, any configuration *c* selected by this policy satisfies $\mathbb {E}\big [\max _i X_i(c)\big ] \le \tau$.

#### Proof

We prove the theorem by induction on the number of requests $n \in \mathbb N$. The base case $n = 0$ is trivial. Now we consider $n>0$. Let *J* be the set of *n* requests. Our algorithm to construct the desired policy *S*(*J*) is the following. Throughout, we fix a truncation threshold $\tau \ge 2\cdot \mathbb {E}[\textsc {Opt}]$.

Let *R* be the random set of requests we recurse on after stopping $\textsc {Opt}(J)$. We first show that indeed $|R |< |J |$, so we can apply induction. Suppose we did not follow $\textsc {Opt}(J)$ to completion because a chosen configuration becomes exceptional (2); denote this event by $\mathcal E$. In this case, there is at least one request for which we have chosen a configuration. Hence, we have $|R| < |J|$, and therefore there is a policy *S* with the required properties by induction.

Suppose now that $\textsc {Opt}(J)$ chooses a configuration $c_j$ for request *j* that is too large (1); denote this event by $\mathcal L$. We have to show that $|R| < |J|$ holds as well. Suppose for the sake of contradiction that *j* was the first request considered by $\textsc {Opt}(J)$. As Opt is w.l.o.g. deterministic, this implies $\mathbb {E}[\textsc {Opt}] \ge \mathbb {E}\big [\max _i X_{ij}(c_j) \big ] > 2 \mathbb {E}[\textsc {Opt}]$, a contradiction. Hence, the desired policy *S* exists by induction.

The maximum load of this policy is at most $\textsc {Opt}(J) + S(R)$, where we set $R = \emptyset$ if no decision results in an exceptional or too large configuration when running $\textsc {Opt}(J)$. In expectation, we have

$$\begin{aligned} \mathbb {E} [S(R)]&= \sum _{J' \subsetneq J} \mathbb {E}[S(R) \mid R = J'] \mathbb {P}[R = J'] = \sum _{ J' \subsetneq J} \mathbb {E} [S(J')] \mathbb {P}[R = J'] \\ {}&\le 2 \sum _{J' \subsetneq J} \mathbb {E} [\textsc {Opt}(J')] \mathbb {P}[R = J] \le 2 \cdot \mathbb {E} [\textsc {Opt}(J)] \mathbb {P}[R \ne \emptyset ]. \end{aligned}$$

In the second equality, we use the fact that the realizations of the remaining requests in *R* are independent of the event $R = J'$. The first inequality uses the inductive hypothesis. The last inequality uses $J' \subseteq J$, so $\mathbb {E} [\textsc {Opt}(J')] \le \mathbb {E} [\textsc {Opt}(J)]$, and $\textsc {Opt}(\emptyset ) = 0$.

Note that on the event $R \ne \emptyset$, we have that $\textsc {Opt}(J)$ chooses a configuration that becomes exceptional or that is too large in expectation. By definition of the policy *S*, the events $\mathcal E$ and $\mathcal L$ are disjoint. By definition of $\mathcal E$, we have $\textsc {Opt}(J)\cdot \mathbbm {1}_\mathcal {E} \ge \tau \cdot \mathbbm {1}_\mathcal {E}$. Observe that the event $\mathcal L$ implies that there is a request $j^*$ with configuration $c^*$ chosen by $\textsc {Opt}(J)$ with $\mathbb {E}\big [\max _i X_{ij^*}(c^*) \big ] \ge \tau$. Since the realization of $\max _i X_{ij^*} (c^*)$ is independent of the choice $c^*$, this implies $\mathbb {E}[\textsc {Opt}\mid \mathcal L ] \ge \mathbb {E}[\max _i X_{ij^*}(c^*) \mid \mathcal L] = \mathbb {E}[\max _i X_{ij^*}(c^*)] \ge \tau$. Thus,

$$\begin{aligned} \mathbb {E}[\textsc {Opt}(J)]&\ge \mathbb P[ \mathcal E] \mathbb {E}[\textsc {Opt}(J) \mid \mathcal E ] + \mathbb P [ \mathcal L] \mathbb {E}[\textsc {Opt}(J) \mid \mathcal L ] \\ {}&\quad \ge \mathbb P[ \mathcal E] \tau + \mathbb P [ \mathcal L] \tau \ge 2 \mathbb P[R \ne \emptyset ] \mathbb {E}[\textsc {Opt}(J)]. \end{aligned}$$

Rearranging yields $\mathbb {P}[R \ne \emptyset ] \le \frac{1}{2}$. Hence, we can bound the expected makespan of policy *S*(*J*) by

$$ \mathbb {E} [\textsc {Opt}(J)] + \mathbb {E} [S(R)] \le \mathbb {E} [\textsc {Opt}(J)] + 2 \mathbb {E} [\textsc {Opt}(J)] \mathbb {P}[R \ne \emptyset ] \le 2 \mathbb {E} [\textsc {Opt}(J)]. $$

The computation for the total expected exceptional load is similar. We let $j \rightarrow c$ denote the event that our policy chooses configuration *c* for request *j*. Then, we can split the exceptional load into two parts based on whether a configuration is chosen by $\textsc {Opt}(J)$ or *S*(*R*)

$$\begin{aligned} \sum _{j=1}^n \sum _{c=1}^{q_j} \Big (\max _i X_{ij}^E(c) \Big ) \cdot \mathbbm {1}_{j \rightarrow c} \\ = \sum _{j=1}^n \sum _{c=1}^{q_j}\Big (\max _i X_{ij}^E(c) \Big ) \cdot \mathbbm {1}_{j \xrightarrow {J} c} + \sum _{j=1}^n \sum _{c=1}^{q_j} \Big (\max _i X_{ij}^E(c) \Big ) \cdot \mathbbm {1}_{j \xrightarrow {R} c}, \end{aligned}$$

where we let $j \xrightarrow {J} c$ and $j \xrightarrow {R} c$ denote the events that configuration *c* is chosen for request *j* by $\textsc {Opt}(J)$ up to the first too large configuration or up to and including the first exceptional configuration, or by *S*(*R*), respectively.

We first bound the former term, corresponding to the configurations chosen in $\textsc {Opt}(J)$. In case of event $\mathcal L$ or if $\textsc {Opt}(J)$ is run to completion, we have $\sum _{j,c} \big (\max _i X_{ij}^E(c) \big ) \cdot \mathbbm {1}_{j \xrightarrow {J} c} = 0$. Otherwise, let $j^* \rightarrow c^*$ be the first (and only) exceptional configuration chosen by $\textsc {Opt}(J)$. Then, $\sum _{j,c} \big (\max _i X_{ij}^E(c) \big ) \cdot \mathbbm {1}_{j \xrightarrow {J} c} = \max _i X_{ij^*}^E(c^*) \le \textsc {Opt}(J)$. Combining and taking expectations yields

$$ \mathbb {E} \bigg [\sum _{j=1}^n \sum _{c=1}^{q_j} \max _i X_{ij}^E(c) \cdot \mathbbm {1}_{j \xrightarrow {J} c}\bigg ] \le \mathbb {E} [\textsc {Opt}(J)]. $$

For the latter term, we condition again on the events $R = J'$ and apply the inductive hypothesis. All exceptional parts are defined with respect to the fixed threshold $\tau \ge 2 \cdot \mathbb {E} [\textsc {Opt}(J)] \ge 2 \cdot \mathbb {E} [\textsc {Opt}(J')]$ for $J' \subset J$. Therefore,

$$\begin{aligned}&\mathbb {E} \bigg [\sum _{j=1}^n \sum _{c=1}^{q_j} \big (\max _i X_{ij}^E(c) \big ) \cdot \mathbbm {1}_{j \xrightarrow {R} c}\bigg ] \\&\quad = \sum _{ J' \subsetneq J} \mathbb {E} \bigg [\sum _{j \in J'} \sum _{c=1}^{q_j} \big (\max _i X_{ij}^E(c) \big ) \cdot \mathbbm {1}_{j \xrightarrow {R} c} \,\Big |\, R = J'\bigg ] \cdot \mathbb {P}[R = J']\\&\quad = \sum _{ J' \subsetneq J} \mathbb {E}\bigg [ \sum _{j \in J'} \sum _{c=1}^{q_j} \big (\max _i X_{ij}^E(c) \big ) \cdot \mathbbm {1}_{j \xrightarrow {J'} c}\bigg ] \cdot \mathbb {P}[R = J']\\&\quad \le 2 \sum _{ J' \subsetneq J} \mathbb {E} [\textsc {Opt}(J')] \cdot \mathbb {P}[R = J']\\&\quad \le 2 \cdot \mathbb {E} [\textsc {Opt}(J)] \cdot \mathbb {P}[R \ne \emptyset ] \le \mathbb {E} [\textsc {Opt}(J)]. \end{aligned}$$

Note that we define $j \xrightarrow {J'} c$ to be the event that the policy $S(J')$ chooses configuration *c* for request *j*. In conclusion, by combining our bounds for these two terms we have

$$ \mathbb {E} \bigg [\sum _{j=1}^n \sum _{c=1}^{q_j} \big (\max _i X_{ij}(c)^E \big ) \cdot \mathbbm {1}_{j \rightarrow c} \bigg ] \le 2 \mathbb {E} [\textsc {Opt}(J)]. $$

To conclude, our constructed policy has expected maximum truncated load and total expected exceptional load both at most $2 \cdot \mathbb {E}[\textsc {Opt}{}]$ (by the above calculations), and it never chooses a configuration with $\mathbb {E}\big [ \max _i X_i(c) \big ] > \tau$ (because we stop running $\textsc {Opt}(J)$ right before it chooses such a configuration, and by induction we subsequently do not as well.) $\square$

Having this near-optimal policy at hand, the upshot is that we can bound our subsequent algorithms with respect to the following LP relaxation ($\textsf {LP}_{\textsf {C}}$) for configuration balancing with stochastic requests. The variable $y_{cj}$ denotes selecting configuration *c* for request *j*. We take our threshold between the truncated and exceptional parts to be $\tau$. Using the natural setting of the *y*-variables as the probabilities of the policy from Theorem 7, it is straight-forward to show that the following LP relaxation has a feasible solution, formalized in Lemma 8. 
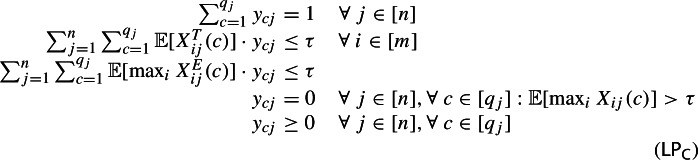


#### Lemma 8

($\textsf {LP}_{\textsf {C}}$) has a feasible solution for any $\tau \ge 2 \cdot \mathbb {E}[\textsc {Opt}]$.

#### Proof

Consider the adaptive policy guaranteed by Theorem 7, say $\mathcal {P}$, and let the events $j \rightarrow c$ be with respect to $\mathcal {P}$. We consider the natural setting of the *y*-variables given this policy: for all *j* and $c \in [q_j]$, we take $y_{cj} = \mathbb {P}(j \rightarrow c)$. It is clear that $\sum _{c=1}^{q_j} y_{cj} = 1$ for all *j* and $0 \le y_{cj} \le 1$ for all *j* and for all $c\in [q_j]$. Moreover, as by Theorem 7 the policy does not select a configuration *c* with $\mathbb {E}\big [ \max _i X_i(c) \big ] > \tau$, the pruning constraints $y_{cj} = 0$ if $\mathbb {E}\big [ \max _i X_i(c) \big ] > \tau$ are also satisfied.

It remains to verify the exceptional and truncated load constraints. For the exceptional constraint, we observe

$$\begin{aligned} \sum _{j=1}^n\sum _{c=1}^{q_j} \mathbb {E}\Big [\max _i X_{ij}^E(c)\Big ] \cdot y_{cj} = \sum _{j=1}^n\sum _{c=1}^{q_j} \mathbb {E}\Big [\max _i X_{ij}^E(c) \cdot \mathbbm {1}_{j \rightarrow c}\Big ] \le 2 \cdot \mathbb {E}[\textsc {Opt}] \le \tau , \end{aligned}$$

where in the first step we use the fact that the decision to choose configuration *c* for request *j* is independent of its realization, and in the second we use the properties of the policy. Similarly, for the truncated constraint for each *i*,

$$ \sum _{j=1}^n\sum _{c=1}^{q_j} \mathbb {E}\big [X_{ij}^T(c)\big ] \cdot y_{cj} = \sum _{j=1}^n\sum _{c=1}^{q_j} \mathbb {E}\big [X_{ij}^T(c) \cdot \mathbbm {1}_{j \rightarrow c}\big ] \le \sum _{j=1}^n\sum _{c=1}^{q_j} \mathbb {E}\big [X_{ij}(c) \cdot \mathbbm {1}_{j \rightarrow c}\big ], $$

where we additionally use the fact that $X^T \le X$ almost surely for any non-negative random variable *X* and truncation threshold. This final expression is exactly the expected load of machine *i* in policy $\mathcal {P}$. To show that all truncated load constraints are satisfied, it suffices to show that the maximum expected load in $\mathcal {P}$ is at most $\tau$. Applying Jensen’s inequality and the properties of $\mathcal {P}$ give

$$\begin{aligned} \max _i \bigg (\sum _{j=1}^n\sum _{c=1}^{q_j} \mathbb {E}\big [X_{ij}(c) \cdot \mathbbm {1}_{j \rightarrow c}\big ] \bigg ) \! \le \! \mathbb {E}\big [\max _i \sum _{j=1}^n\sum _{c=1}^{q_j} X_{ij}(c) \cdot \mathbbm {1}_{j \rightarrow c}\big ]\\ =\! \mathbb {E}[\mathcal {P}] \le 2 \cdot \mathbb {E}[\textsc {Opt}] \! \le \! \tau , \end{aligned}$$

as required. $$\square $$

### Offline setting

Our offline algorithm is the natural randomized rounding of ($\textsf {LP}_{\textsf {C}}$). First, we use a standard guess-and-double approach to find a truncation threshold $\tau$ such that ($\textsf {LP}_{\textsf {C}}$) is feasible. Then once we have a feasible solution $y^*$, for each request *j*, we choose configuration *c* as configuration $c_j$ independently with probability $y_{cj}^*$; see Algorithm 2 for the formal algorithm.

**Algorithm 2:**
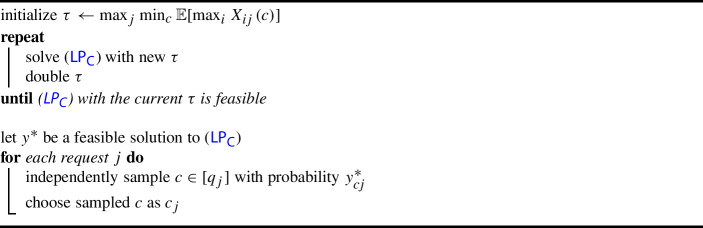
Offline Configuration Balancing with Stochastic Requests

To analyze Algorithm 2, the contribution of the truncated parts to the makespan is bounded by the following inequality, which we prove in Appendix A.

#### Lemma 9

Let $S_1, \dots , S_m$ be sums of independent RVs bounded in $[0,\tau ]$ for some $\tau > 0$ such that $\mathbb {E} [S_i] \le \tau$ for all $i \in [m]$. Then, $\mathbb {E} [\max _i S_i] = O\big (\frac{\log m}{\log \log m}\big )\tau$.

To bound the contribution of the exceptional parts, we use (1), i.e., the total expected exceptional load. The analysis of Algorithm 2 is summarized in the next lemma.

#### Lemma 10

Algorithm 2 is a randomized algorithm that solves ($\textsf {LP}_{\textsf {C}}$) for at most $O\Big (\log \Big ( \frac{\sum _j \max _c \mathbb {E}[\max _i X_{ij}(c)]}{\max _j \min _c \mathbb {E}[\max _i X_{ij}(c)]} \Big )\Big )$-many truncation thresholds and that outputs a non-adaptive policy that $O\big (\frac{\log m}{\log \log m}\big )$-approximates the optimal adaptive policy.

#### Proof

By Lemma 8, we are guaranteed that ($\textsf {LP}_{\textsf {C}}$) is feasible as soon as $\tau \ge 2 \cdot \mathbb {E}[\textsc {Opt}]$. Note that our intial guess of $\tau$ is $\max _j \min _c \mathbb {E}[\max _i X_{ij}(c)]$, which is a trivial lower bound on $\mathbb {E}[\textsc {Opt}]$. A crude upper bound on $\mathbb {E}[\textsc {Opt}]$ is $\sum _j \max _c \mathbb {E}[\max _i X_{ij}(c)]$, so we are guaranteed to double $\tau$ at most $O \Big (\log \Big ( \frac{\sum _j \max _c \mathbb {E}[\max _i X_{ij}(c)]}{\max _j \min _c \mathbb {E}[\max _i X_{ij}(c)]} \Big ) \Big )$-times, as required.

Further, since our initial $\tau$ is at most $\mathbb {E}[\textsc {Opt}]$ and by Lemma 8, our final $\tau$ satisfies $\tau \le 4 \cdot \mathbb {E}[\textsc {Opt}]$. Thus, to show the desired approximation guarantee, it suffices to show that Algorithm 2 returns a non-adaptive policy with expected makespan $O\big (\frac{\log m}{\log \log m}\big ) \cdot \tau$.

We let $j \rightarrow c$ denote the event that our algorithm chooses configuration *c* for request *j*. Thus, the truncated load on resource *i* can be written as

$$\begin{aligned} L_i = \sum _{j=1}^{n} \bigg ( \sum _{c=1}^{q_j} X^T_{ij}(c) \cdot \mathbbm {1}_{j \rightarrow c} \bigg ). \end{aligned}$$

Note that the random variables $\sum _{c=1}^{q_j} X_{ij}^T(c) \cdot \mathbbm {1}_{j \rightarrow c}$ are independent for different *j* because the $X_{ij}$ are and we sample the configuration $c_j$ independently for each *j*. Further, they are bounded in $[0,\tau ]$ by truncation. With the constraints of ($\textsf {LP}_{\textsf {C}}$), we can bound the expectation by

$$\begin{aligned} \mathbb {E}[L_i] = \sum _{j=1}^{n} \sum _{c=1}^{q_j} \mathbb {E}\Big [X_{ij}^T(c) \cdot \mathbbm {1}_{j \rightarrow c}\Big ] = \sum _{j=1}^{n} \sum _{c=1}^{q_j} \mathbb {E}\Big [X_{ij}^T(c)\Big ] \cdot y^*_{cj} \le \tau , \end{aligned}$$

where we used the independence of $\mathbbm {1}_{j \rightarrow c}$ and $X_{ij}(c)$ in the second equality. By Lemma 9, $\mathbb {E}[\max _i L_i] = O\big (\frac{ \log m}{\log \log m}\big ) \cdot \tau$. Using (1) we upper bound the total expected exceptional load by

$$\begin{aligned} \mathbb {E}\bigg [\max _{1\le i \le m} \sum _{j=1}^{n} \sum _{c=1}^{q_j} X^E_{ij}(c) \cdot \mathbbm {1}_{j \rightarrow c} \bigg ]&\le \sum _{j=1}^{n} \sum _{c=1}^{q_j} \mathbb {E}\Big [ \max _{1\le i \le m} X^E_{ij}(c) \cdot \mathbbm {1}_{j \rightarrow c} \Big ] \\&= \sum _{j=1}^{n} \sum _{c=1}^{q_j} \mathbb {E}\Big [\max _{1\le i \le m} X^E_{ij}(c)\Big ] \cdot y_{cj}^* \le \tau . \end{aligned}$$

Combining our bounds for the truncated and exceptional parts completes the proof. The expected makespan of our algorithm is given by

$$\begin{aligned} \mathbb {E}\Big [\max _{1\le i \le m} \sum _{j=1}^{n} \sum _{c=1}^{q_j} X_{ij}(c) \cdot \mathbbm {1}_{j \rightarrow c} \Big ]&\le \mathbb {E}\Big [\max _{1\le i \le m} L_i \Big ] + \mathbb {E}\Big [\max _{1\le i \le m} \sum _{j=1}^{n} \sum _{c=1}^{q_j} X^E_{ij}(c) \cdot \mathbbm {1}_{j \rightarrow c} \Big ] \\&= O\Big (\frac{ \log m}{\log \log m}\Big ) \cdot \tau , \end{aligned}$$

as required. $$\square $$

If the configurations are given explicitly as part of the input, then ($\textsf {LP}_{\textsf {C}}$) can be solved in polynomial time, and we do this for polynomially-many truncation thresholds. Thus, Algorithm 2 runs in polynomial time. Hence, the $O\big (\frac{\log m}{\log \log m}\big )$-approximate non-adaptive policy for configuration balancing with stochastic requests (Theorem 1) follows from the lemma.

### Online setting

We now consider online configuration balancing where *n* stochastic requests arrive online one-by-one, and for each request, one configuration has to be irrevocably selected before the next request appears. We present a non-adaptive online algorithm that achieves a competitive ratio of $O(\log m)$, which is best possible due to the lower bound of $\Omega (\log m)$ [6].

By a standard guess-and-double scheme, we may assume that we have a good guess of $\mathbb {E}[\textsc {Opt}]$. The proof is analogous to [5]; see Appendix D for details.

#### Lemma 11

Given an instance of online configuration balancing with stochastic requests, suppose there exists an online algorithm that, given parameter $\lambda > 0$, never creates an expected makespan more than $\alpha \cdot \lambda$, possibly terminating before handling all requests. Further, if the algorithm terminates prematurely, then it certifies that $\mathbb {E}[\textsc {Opt}] > \lambda$. Then, there exists an $O(\alpha )$-competitive algorithm for online configuration balancing with stochastic requests. Further, the resulting algorithm preserves non-adaptivity.

We will build on the same technical tools as in the offline case. In particular, we wish to compute a non-adaptive assignment online with small expected truncated load on each resource and small total expected exceptional load. To achieve this, we generalize the greedy potential function approach of [5]. Our two new ingredients are to treat the exceptional parts of a request’s configuration as a resource requirement for an additional, artificial resource and to compare the potential of our solution directly with a *fractional* solution to ($\textsf {LP}_{\textsf {C}}$).

Now we describe our potential function, which is based on an exponential/soft-max function. Let $\lambda$ denote the current guess of the optimum as required by Lemma 11. We take $\tau = 2 \lambda$ as our truncation threshold. Given load vector $L \in \mathbb {R}^{m+1}$, our potential function is

$$ \phi (L) = \sum _{i=0}^m (3/2)^{ L_i / \tau }. $$

For $i\in [m]$, we ensure the *i*th entry of $L$ is the *expected* truncated load on resource *i* and use the 0th entry as a virtual resource that is the total expected exceptional load. For any request *j*, let $L_j$ be the expected load vector after handling the first *j* requests, with $L_{ij}$ denoting its *i*th entry. Let $L_{i0}:= 0$ for all *i*. Upon arrival of request *j*, our algorithm tries to choose the configuration $c_{j} \in [q_{j}]$ that minimizes the increase in potential; see Algorithm 3.

**Algorithm 3:**
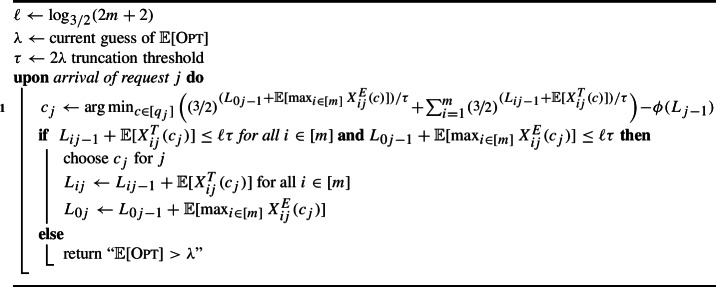
Online Configuration Balancing with Stochastic Requests

To analyze this algorithm, we compare its makespan with a solution to ($\textsf {LP}_{\textsf {C}}$). This LP has an integrality gap of $\Omega \big (\frac{\log m}{\log \log m}\big )$, which follows immediately from the path assignment LP for virtual circuit routing [4]. Hence, a straightforward analysis of Algorithm 3 comparing to a rounded solution to ($\textsf {LP}_{\textsf {C}}$) gives an assignment with expected truncated load per machine and total expected exceptional load $O\big (\log m \cdot \frac{\log m}{\log \log m}) \cdot \mathbb {E}[\textsc {Opt}]$. To get a tight competitive ratio of $O(\log m)$, we avoid the integrality gap by comparing to a *fractional* solution to ($\textsf {LP}_{\textsf {C}}$), and we use a slightly different inequality than Lemma 9 for the regime where the mean of the sums is larger than the increments by at most a $O(\log m)$-factor. The particular maximal inequality is the following, which we prove in Appendix A.

#### Lemma 12

Let $S_1, \ldots , S_m$ be sums of independent RVs bounded in $[0,\tau ]$ for $\tau >0$ such that $\mathbb {E}[S_i] \le O(\log m) \tau$ for all $1 \le i \le m$. Then, $\mathbb {E}[\max _i S_i] \le O(\log m) \tau$.

We give the guarantee for Algorithm 3, which implies the $O(\log m)$-competitive algorithm for online configuration balancing with stochastic requests.

#### Lemma 13

Suppose the minimizing configuration in Line 1 can be found in polynomial time. Then Algorithm 3 runs in polynomial time; it is deterministic, non-adaptive and correctly solves the subproblem of Lemma 11 for $\alpha = O(\log m)$.

#### Proof

The running time of Algorithm 3 is clear. It remains to show that the algorithm creates expected makespan at most $O(\log m) \lambda$ or correctly certifies $\mathbb {E}[\textsc {Opt}] > \lambda$ if it terminates prematurely.

We first show the former. By definition, upon termination of the algorithm after, say $n'$ requests, the expected truncated load on each resource and total expected exceptional load are both at most $O(\log m) \cdot \lambda$. Let the configuration choices $c_j$ be with respect to our algorithm. We can split the makespan into the contributions by the truncated and exceptional parts.

$$ \mathbb {E}\bigg [\max _{1 \le i \le m } \sum _{j=1}^{n'} X_{ij}(c_j) \bigg ] \le \mathbb {E}\bigg [\max _{1 \le i \le m} \sum _{j=1}^{n'} X_{ij}^T(c_j) \bigg ] + \mathbb {E}\bigg [\max _{1 \le i \le m} \sum _{j=1}^{n'} X_{ij}^E(c_j) \bigg ]. $$

Each truncated part is bounded in $[0, 2\lambda ]$ and each resource has expected truncated load at most $O(\log m) \lambda$. By Lemma 12, we can bound the contribution of the truncated parts by $\mathbb {E}\big [\max _{1 \le i \le m} \sum _{j=1}^{n'} X_{ij}^T(c_j) \big ] = O(\log m) \lambda$.

For the exceptional parts, applying (1) gives

$$ \mathbb {E}\bigg [\max _{1 \le i \le m} \sum _{j=1}^n X_{ij}^E(c_j) \bigg ] \le \mathbb {E}\bigg [\sum _{j=1}^n \max _{1 \le i \le m} X_{ij}^E(c_j) \bigg ] \le O(\log m) \lambda . $$

Combining both bounds gives that the expected makespan is at most $O(\log m) \lambda$, as required.

It remains to show that if $\mathbb {E}[\textsc {Opt}] \le \lambda$, then the algorithm successfully assigns all requests. We do so by bounding the increase in the potential function. Note that if $\mathbb {E}[\textsc {Opt}] \le \lambda$, then ($\textsf {LP}_{\textsf {C}}$) is feasible for our choice of $\tau = 2 \lambda$ by Lemma 8. Thus, let $(y_{cj})$ be a feasible solution to ($\textsf {LP}_{\textsf {C}}$). For simplicity, let $x_{0j}(c):= \mathbb {E}[\max _{1\le i \le m} X_{ij}^E(c)]$ be the exceptional part of configuration *c* and $x_{ij}(c):= \mathbb {E}[X_{ij}^T(c)]$ its truncated part on resource *i*.

For each request *j*, as Algorithm 3 chooses a configuration $c_j$ minimizing the increase in $\Phi$,

$$\begin{aligned} \phi (L_{j-1} + x_j(c_j)) - \phi (L_{j-1})&\le \phi (L_{j-1} + x_j(c)) - \phi (L_{j-1}) \end{aligned}$$

for all configurations $c \in [q_j]$. As $\sum _{c=1}^{q_j} y_{cj} = 1$ by feasibility of $(y_{cj})$, this implies

2 $$\begin{aligned} \phi (L_{j-1} + x_j(c_j)) - \phi (L_{j-1})&\le \sum _{c=1}^{q_j} y_{cj} \phi (L_{j-1} + x_{j}(c)) - \phi (L_{j-1}). \end{aligned}$$

We recall that $L_0 = 0 \in \mathbb {R}^{m+1}$. We bound the increase in potential incurred by Algorithm 3:

$$\begin{aligned} \phi (L_n) - \phi (L_0)&= \sum _{j=1}^n \sum _{i=0}^m (3/2)^{L_{ij-1}/\tau } \Big ( (3/2)^{x_{ij}(c_j)/\tau } - 1 \Big ) \\&\le \sum _{j=1}^n \sum _{c=1}^{q_j} y_{cj} \sum _{i=0}^m (3/2)^{L_{ij-1}/\tau } \Big ( (3/2)^{x_{ij}(c)/\tau } - 1 \Big ) \\&\le \sum _{i=0}^m (3/2)^{L_{in}/\tau } \sum _{j=1}^n \sum _{c=1}^{q_j} y_{cj} \Big ( (3/2)^{x_{ij}(c)/\tau } -1 \Big ), \end{aligned}$$

where the first inequality holds due to (2), and the second inequality holds because the load on machine *i* only increases over time. Standard estimates of $e^z$ give $(3/2)^z - 1 \le (1/2) z$ for $z \in [0,1]$. As $y_{cj}$ is feasible, we know that $x_{ij}(c) \le \tau$ for all *c* with $y_{cj} > 0$. Hence,

$$\begin{aligned} \phi (L_n) - \phi (L_0)&\le \sum _{i=0}^m (3/2)^{L_{in}/\tau } \sum _{j=1}^n \sum _{c=1}^{q_j} y_{cj} \frac{x_{ij}(c)}{2\tau } \end{aligned}$$

Using that $y_{cj}$ is feasible and satisfies $\sum _{j=1}^n \sum _{c=1}^{q_j} x_{ij}(c) \cdot y_{cj} \le \tau$ for resource $i = 0$ by the exceptional constraint and for all resources $i \in [m]$ by the truncated constraints, we get

$$\begin{aligned} \phi (L_n) - \phi (L_0)&\le (1/2) \sum _{i=0}^m (3/2)^{L_{in}/\tau } = (1/2) \phi (L_n). \end{aligned}$$

After rearranging, we have $\phi (L_n) \le 2 \phi (L_0) = 2(m+1)$ by choice of $L_0$. Taking logarithms and using that $\log _{3/2}(z)$ is monotonically increasing, we conclude that $\max _{0 \le i \le m} L_{in} \le \log _{3/2} (2\,m + 2) \tau .$ Note that we chose $\ell = \log _{3/2} (2m + 2)$ implying that Algorithm 3 never fails if $\mathbb {E}[\textsc {Opt}] \le \lambda$. $\square$

#### Proof of Theorem 1

Note that the configurations are given explicitly, implying that both, Algorithms 2 and 3, run in polynomial time. Hence, Lemma 10 gives the $O(\frac{\log m}{\log \log m})$-approximate offline algorithm and Lemmas 11 and 13 give the $O(\log m)$-approximate online algorithm. $\square$

## Unrelated load balancing and virtual circuit routing

In this section, we apply our algorithms for configuration balancing to stochastic load balancing on unrelated machines (Theorem 2) as well as stochastic virtual circuit routing (Theorem 3).

### Unrelated load balancing with stochastic jobs

We recall that in load balancing on unrelated machines with stochastic jobs, we have *m* machines and *n* jobs such that the size of job *j* on machine *i* is a random variable $X_{ij}$. These $X_{ij}$s are independent across jobs. This is a special case of configuration balancing with stochastic requests by taking *m* resources (corresponding to the *m* machines) and *n* requests such that each request *j* has *m* possible configurations, one for each machine choice job *j* has. Precisely, we define the configurations $c\in [m]$ for job *j* by setting

3 $$\begin{aligned} X_{ij}(c) = {\left\{ \begin{array}{ll} X_{cj} &{} if i = c, \\ 0 &{} otherwise . \end{array}\right. } \end{aligned}$$

#### Proof of Theorem 2

Each request has *m* possible configurations, so the total size of the resulting configuration balancing instance is polynomial. Thus, we may assume the configurations are given explicitly. Hence, Theorem 1 immediately gives a *randomized* $O(\frac{\log m}{\log \log m})$-approximation offline and $O(\log m)$-approximation online for load balancing on unrelated machines with stochastic jobs.

However, to obtain a deterministic offline algorithm, we can de-randomize Algorithm 2 for this special case because here, ($\textsf {LP}_{\textsf {C}}$) is equivalent to the generalized assignment LP considered by Shmoys and Tardos, which has a constant-factor rounding algorithm [7]. $\square$

### Routing with stochastic demands

Virtual circuit routing is another special case of configuration balancing: Given any instance of the former, which consists of a directed graph with *m* edges and *n* source-sink pairs, the resulting configuration balancing instance has *m* resources, one for each edge, and *n* requests, one for each source-sink pair, such that there is one configuration per source-sink path in the graph.

Note that unlike load balancing, where a request has at most *m* configurations, for routing, a request can have exponentially many configurations. Thus, to obtain polynomial-time algorithms for routing, we cannot explicitly represent all configurations. In particular, to prove Theorem 3 in the offline setting, it suffices to show how to solve ($\textsf {LP}_{\textsf {C}}$) efficiently in the special case of routing. This is because, if the request distributions are given explicitly, then by Lemma 10, we solve ($\textsf {LP}_{\textsf {C}}$) polynomially-many times. Similarly, to prove Theorem 3 in the online setting, we need to show how to find the configuration (i.e. the path) that minimizes the increase in the potential $\Phi$.

#### Offline routing: solving ($\textsf {LP}_{\textsf {C}}$)

We first re-write ($\textsf {LP}_{\textsf {C}}$) for routing. Recall that $\tau$ is the truncation threshold. For a request *j*, let $E_j$ be the set of all edges with $\mathbb {E}[X_{ej}] \le \tau$ and let $\mathcal P_j$ be the collection of all $a_j$-$b_j$-paths in the auxiliary graph $G_j = (V,E_j)$. For each request *j* and path $P \in \mathcal P_j$, the variable $y_{Pj}$ denotes the decision to route request *j* along path *P*. Thus, we want to solve the following path assignment LP. 
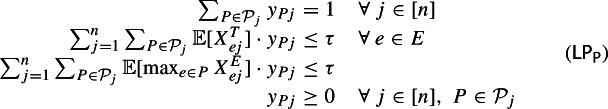


To see that ($\textsf {LP}_{\textsf {P}}$) is equivalent to ($\textsf {LP}_{\textsf {C}}$), note that the pruning constraints of the latter ensure that any configuration/path *P* with $\mathbb {E}\big [ \max _{e \in P} X_{ej} \big ] > \tau$ will not be selected. Re-writing gives $\mathbb {E}\big [ \max _{e \in P} X_{ej} \big ] = \mathbb {E}\big [ \max _{e \in P} \frac{1}{s_e} X_j \big ]= \max _{e \in P} \mathbb {E}[X_{ej}]$. Thus, the pruning constraint ensures that no edge with $\mathbb {E}[X_{ej}] > \tau$ will be selected. This is exactly encoded by the set of feasible paths $\mathcal {P}_j$.

Note that ($\textsf {LP}_{\textsf {P}}$) has an exponential number of variables. For classical congestion minimization problems, the path-assignment LP formulation is equivalent to a flow formulation [4], which can be solved optimally in polynomial time using results about flows in networks. In our case, however, we additionally have the third constraint (the exceptional load constraint) in the LP, which does not allow for a straight-forward equivalent flow formulation. Therefore, we use LP duality in order to solve it efficiently: We give a separation oracle for its dual LP. For obtaining the dual, we add the trivial objective to maximize $0^T y$ to ($\textsf {LP}_{\textsf {P}}$). Hence, the dual of ($\textsf {LP}_{\textsf {P}}$), with variables $\beta _j$ for $j \in J$, $\gamma _e$ for $e \in E$ and $\delta$, is 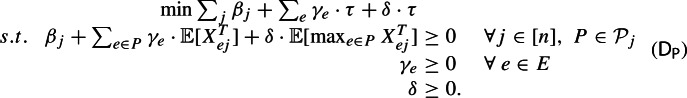


For solving ($\textsf {D}_{\textsf {P}}$), consider a request *j* and a path $P \in \mathcal P_j$. The expected exceptional part of routing *j* along *P* is $\mathbb {E}\big [\max _{e \in P} X_{ej} ^E \big ] = \max _{e \in P} \mathbb {E}\big [\big ({X_j}/{s_e}\big )^E\big ]$, which is the expected exceptional part of the smallest-capacity edge $\bar{e}_j$ along the path. Given this edge $\bar{e}_j$, a particular choice of the dual variables is feasible for the first constraint of ($\textsf {D}_{\textsf {P}}$) if and only if

$$\begin{aligned} \min _{P \in \mathcal P_j \,: \, \min _{e \in P} s_e \ge s_{\bar{e}_j} } \bigg ( \sum _{e \in P} \gamma _e \cdot \mathbb {E}\big [X_{ej}^T\big ] + \delta \cdot \mathbb {E}\big [\max _{e \in P} X_{ej}^T\big ] \bigg ) \ge -\beta _j \quad \forall ~ j. \end{aligned}$$

Hence, for each request *j*, it remains to find a path $P \in \mathcal P_j$ that is minimal w.r.t. edge weights $\gamma _e \cdot \mathbb {E}[X_{ej}^T]$. By letting every edge $\bar{e}$ be the smallest-capacity edge and removing any smaller-capacity edges from the graph, this becomes a shortest $a_j$-$b_j$ path problem.

**Algorithm 4:**
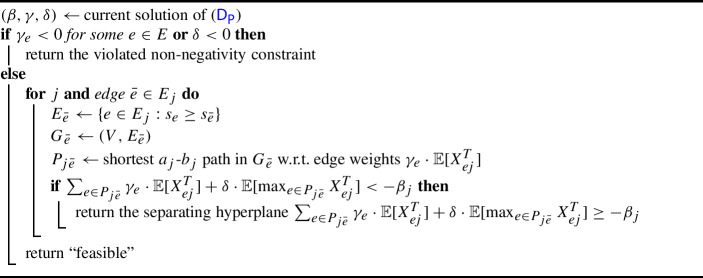
Separation oracle for ($\textsf {D}_{\textsf {P}}$)

##### Lemma 14

Algorithm 4 is a polynomial time separation oracle for ($\textsf {D}_{\textsf {P}}$).

##### Proof

Since shortest paths can be found in polynomial time [44], Algorithm 4 indeed runs in polynomial time. Further, if there is an edge $e \in E$ with $\gamma _e < 0$ or if $\delta <0$, then Algorithm 4 correctly outputs the violated constraint.

It remains to consider the case when $\gamma , \delta \ge 0$. In this case, we need to show for each request *j* that we find some $\bar{e} \in E$ such that $P_{j \bar{e}}$ achieves $\min _{P \in \mathcal P_j} \big ( \sum _{e \in P} \gamma _e \cdot \mathbb {E}[X_{ej}^T] + \delta \cdot \mathbb {E}[\max _{e \in P} X_{ej}^T] \big )$.

Consider any request *j* such that the minimum is achieved by some $a_j$-$b_j$ path $P^*$. Let $e^*$ be the smallest-capacity edge along $P^*$. We claim for the correct guess $\bar{e} = e^*$, $P_{j \bar{e}}$ achieves the minimum. To see this, observe that $P^*$ is a $a_j$-$b_j$ path in the graph $G_{\bar{e}}$, so the algorithm will choose $P_{j \bar{e}}$ with $\sum _{e \in P_{j\bar{e}}} \gamma _e \cdot \mathbb {E}[X_{ej}^T] \le \sum _{e \in P^*} \gamma _e \cdot \mathbb {E}[X_{ej}^T]$ by definition of the edge weights. Further, we have $\delta \cdot \mathbb {E}[\max _{e \in P_{j\bar{e}}} X_{ej}^T] \le \delta \cdot \mathbb {E}[\max _{e \in P^*} X_{ej}^T]$, because the latter maximum is achieved by edge $\bar{e}$, and by definition of the residual graph, $P_{j \bar{e}}$ cannot use any edges with smaller capacity than $\bar{e}$. Combining these two bounds shows that $P_{j \bar{e}}$ achieves the minimum. This in turn implies that Algorithm 4 returns a constraint violated by $(\beta , \gamma , \delta )$ if such a constraint exists. $\square$

#### Online routing: minimizing increase in the potential

For online virtual circuit routing, we assume that a sequence of source sink-pairs $(a_j, b_j)$ for $j \in [n]$ arrive online. To implement Algorithm 3 efficiently, given a load vector $L= (L_0, \dots , L_m)$ and source-sink pair $(a_j, b_j)$ with random demand $X_j \ge 0$, we need to choose a $a_j$-$b_j$ path in *G* that minimizes the increase in $\Phi$ with respect to some fixed truncation threshold $\tau$. Recall that we index the edges of *G* by $1, \dots , m$, while $L_0$ is the load of an additional, artificial resource that captures the total expected exceptional load.

As in the offline setting, the expected exceptional part of the configuration corresponding to choosing a particular $a_j$-$b_j$ path is the expected exceptional part of the smallest-capacity edge along the path. Thus, the increase in potential due to choosing a path *P* is

$$ \big ( (3/2)^{ (L_{0} + ( \max _{e \in P} \mathbb {E}[X_{ej}^E]) / \tau } - (3/2)^{ L_{0} / \tau }\big ) + \sum _{e \in P} \big ( (3/2)^{ ( L_{e} + \mathbb {E}[X_{ej}^T]) / \tau } - (3/2)^{ L_{e} / \tau }\big ). $$

The first term is the increase due to the exceptional part (the smallest-capacity edge along *P*), and the remaining terms are the per-edge contributions due to the truncated parts. Analogously to the previous section, we only consider edges in $E_j = \{e \in E: \mathbb {E}[X_{ej}]\le \tau \}$, guess the smallest-capacity edge, and solve a shortest $a_j$-$b_j$ path problem to find the minimizing path; see Algorithm 5.

**Algorithm 5:**
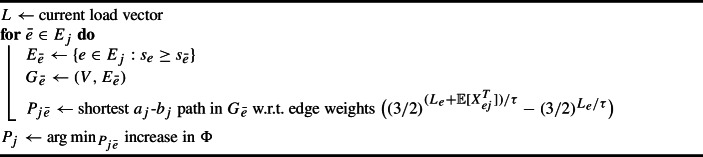
Minimizing increase in $\Phi$ for virtual circuit routing

##### Lemma 15

Given a request *j* and load vector *L*, Algorithm 5 returns in polynomial time a path $P_j$ that minimizes the increase in $\Phi$.

##### Proof

It is clear that Algorithm 5 runs in polynomial time. To see that it also finds a path that minimizes the increase in $\Phi$, let $P_j^*$ be such an optimal path with smallest-capacity edge $e^*$. For the correct guess $\bar{e} = e^*$, $P_j^*$ is a $a_j$-$b_j$ path in the graph $G_{\bar{e}}$. Thus, Algorithm 5 chooses $P_{j\bar{e}}$ such that the per-edge contributions due to the truncated parts of $P_{j\bar{e}}$ are at most those due to $P_j^*$ by definition of the edge weights. Further, the exceptional part of $P_{j\bar{e}}$ is at most that of $P_j^*$, because $P_{j \bar{e}}$ does not use any edges with capacity smaller than $s_{\bar{e}}$ by definition of the graph $G_{\bar{e}}$. We conclude that $P_{j\bar{e}}$ is also a $a_j$-$b_j$ path that minimizes the increase in $\Phi$. $\square$

##### Proof of Theorem 3

For offline virtual circuit routing, Lemma 14 guarantees that ($\textsf {LP}_{\textsf {P}}$) can be solved optimally in polynomial time by LP duality. Thus, Lemma 10 implies that Algorithm 2 runs in polynomial time and achieves a maximum congestion of $O\big (\frac{\log m}{\log \log m}\big ) \mathbb {E}[\textsc {Opt}]$. For the online problem, Lemma 15 guarantees that, for each request *j*, a path $P_j$ that minimizes the increase in the potential function $\Phi$ is found in polynomial time. Thus, Lemma 10 implies that Algorithm 3 runs in polynomial time and guarantees a maximum congestion of $O(\log m)\mathbb {E}[\textsc {Opt}]$. $\square$

## Load balancing on related machines

In this section, we improve on Theorem 2 in the special case of related machines, where each machine *i* has a speed parameter $s_i > 0$ and each job *j* an independent size $X_j$ such that $X_{ij} = \frac{X_j}{s_i}$. Recall that we gave a non-adaptive $O\big (\frac{\log m}{\log \log m}\big )$-approximation for unrelated machines. However, the adaptivity gap is $\Omega \big (\frac{\log m}{\log \log m}\big )$ even for load balancing on identical machines where every machine has the same speed. Thus, to improve on Theorem 2, we need to use adaptivity.

The starting point of our improved algorithms is the same non-adaptive assignment for unrelated-machines load balancing. However, instead of non-adaptively assigning job *j* to the specified machine *i*, we adaptively assign *j* to the least loaded machine with similar speed to *i*. We formalize this idea and briefly explain the algorithms for offline and online load balancing on related machines.

### Machine smoothing

In this part, we define a notion of *smoothed machines*. We show that by losing a constant factor in the approximation ratio, we may assume that the machines are partitioned into at most $O(\log m)$ groups such that machines within a group have the same speed and the size of the groups shrinks geometrically. Thus, by “machines with similar speed to *i*,” we mean machines in the same group.

Formally, we transform an instance $\mathcal I$ of load balancing on *m* related machines with stochastic jobs into an instance $\mathcal I_s$ with so-called “smoothed machines” and the same set of jobs with the following three properties:

(i) The machines are partitioned into $m' = O(\log m)$ groups such that group *k* consists of $m_k$ machines with speed exactly $s_k$ such that $s_1< s_2< \dots < s_{m'}$.
(ii) For all groups $1 \le k < m'$, we have $m_k \ge \frac{3}{2} m_{k+1}$.
(iii) $$\textsc {Opt}(\mathcal I_s) {\le 12 \cdot \textsc {Opt}(\mathcal I)} = O( \textsc {Opt}(\mathcal I))$$.

To this end, we suitably decrease machine speeds and delete machines from the original instance $\mathcal I$. Our algorithm is the following.

**Algorithm 6:**
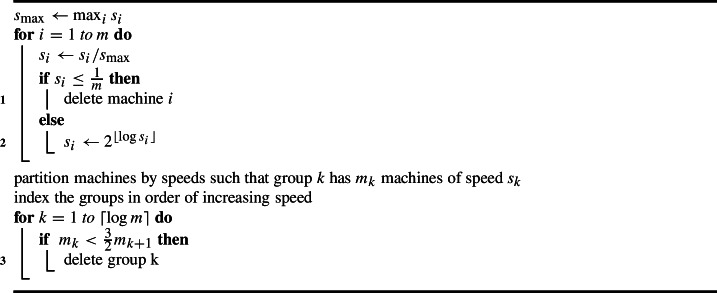
Machine Smoothing

We show that this algorithm creates the desired smoothed machines instance.

#### Lemma 16

There is an efficient algorithm that, given an instance $\mathcal I$ of load balancing with *m* related machines and stochastic jobs, computes an instance $\mathcal I_s$ of smoothed machines with the same set of jobs satisfying Properties (i) to (iii).

#### Proof

It is clear that the algorithm is efficient and outputs $\mathcal {I}_s$ satisfying (i) and (ii). It remains to show (iii). To do so, we show that each step increases Opt by only a constant factor.

Recall that Opt is an adaptive policy, so when Opt decides to schedule job *j* on machine *i*, it immediately learns the realized value of $X_{ij}$, or equivalently $X_j = s_i \cdot X_{ij}$ on related machines. Hence, we may assume that the next scheduling decision of Opt is completely determined by the previously realized $X_j$’s. In particular, if Opt decides to schedule *j* on machine *i*, then we can modify Opt by scheduling *j* on some other machine $i'$ and leaving the subsequent decisions (which in general depend on $X_j$) unchanged. Similarly, if we modify the speed of machine *i*, then this does not affect subsequent scheduling decisions.

1. Let $\textsc {Opt}= \textsc {Opt}(\mathcal {I})$ denote the initial optimal policy. Consider the following adaptive policy for the instance after step 1: If Opt schedules *j* on *i* such that *i* is not deleted, then we also schedule *j* on *i*. Otherwise, Opt schedules *j* on *i* such that *i* is deleted for being too slow. Then we schedule *j* on the fastest machine. For every realization of job sizes, the modified policy only increases the load of the fastest machine. We delete at most $m-1$ machines each having speed at most $\frac{1}{m}$. Thus, we schedule all jobs assigned to these machines on a machine that is at least *m* times faster. The increase in load on the fastest machine is thus at most $(m-1) \cdot \frac{\textsc {Opt}}{m} \le \textsc {Opt}$. After deleting all slow machines, all machine speeds are in $\big (\frac{1}{m}, 1 \big ]$.
2. We decrease the speed of each machine by at most a factor 2, so the makespan of the optimal policy increases by at most a factor 2. Further, after rounding down the machine speeds, there are at most $\lceil \log m \rceil$ distinct speeds and thus groups.
3. First, we note that we keep at least one group, namely the fastest one. Consider any group *k* that is deleted, and let $k' > k$ be the fastest subsequent group that is kept. Because we delete all groups between *k* and $k'$, we have $m_k < \big (\frac{3}{2}\big )^{k' - k} \cdot m_{k'}$. Further, because all groups have distinct speeds that differ by at least a factor 2, we also have $s_{k'} \ge 2^{k' - k} \cdot s_k$. Let $\textsc {Opt}'$ be the optimal policy after step 2. By the above two steps, we have $\textsc {Opt}' \le 2 \cdot (\textsc {Opt}+ \textsc {Opt}) = 4 \cdot \textsc {Opt}$. We re-assign the jobs that $\textsc {Opt}'$ schedules on group *k* to group $k'$ as follows. Because $m_k < \big (\frac{3}{2}\big )^{k' - k} \cdot m_{k'}$, we fix a mapping from the machines in group *k* to those of $k'$ such that each machine in $k'$ is mapped to by at most $\big (\frac{3}{2}\big )^{k' - k}$ machines in *k*. Then, when $\textsc {Opt}'$ schedules a job on a machine in group *k*, we instead schedule it on the machine it maps to in group $k'$. This completes the description of our modified policy. To bound the makespan, consider any machine *i* in a kept group $k'$. We upper-bound the increase in load on *i* due to re-assignments from slower deleted groups. For any deleted group $k < k'$, at most $\big (\frac{3}{2}\big )^{k' - k}$ machines from group *k* map to *i*. Each such machine in group *k* under policy $\textsc {Opt}'$ has load at most $\textsc {Opt}'$. However, recall that *i* is at least a $2^{k'-k}$-factor faster than any machine in group *k*, so the increase in load on machine *i* due to deleted machines from group $k < k'$ is at most $\big (\frac{3}{2}\big )^{k'-k} \cdot 2^{-(k' -k)} \cdot \textsc {Opt}= \big (\frac{3}{4}\big )^{k' -k} \cdot \textsc {Opt}$. Summing over all $k < k'$, the total increase in load on a machine in group $k'$ is at most
  $$\sum _{k < k'} \big (\frac{3}{4}\big )^{k' -k} \cdot \textsc {Opt}' = \le \sum _{k = 1}^\infty \big (\frac{3}{4}\big )^k \cdot \textsc {Opt}' = 3 \cdot \textsc {Opt}'.$$
  We conclude, the makespan of our final solution after 3 is at most $\textsc {Opt}' + 3 \cdot \textsc {Opt}' \le 12 \cdot \textsc {Opt}$, as required.

$$\square $$

To summarize, by losing a constant-factor in our final approximation ratio, we may assume we are working with an instance of smoothed machines. Looking ahead, if our non-adaptive policy assigns job *j* to machine *i*, then we instead adaptively assign *j* to the least-loaded machine in the group containing machine *i*. We will use the properties of smoothed machines to show that this leads to a *O*(1)-approximation offline and $O(\log \log m)$-approximation online.

A similar idea for machine smoothing has been employed by Im et al. [34] for deterministic load balancing on related machines. In their approach, they ensure that the *total processing power* of the machines in a group decreases geometrically rather than the number of machines.

### Offline setting

We run Algorithm 2 on the configuration balancing instance defined by the load balancing instance with smoothed machines. Given a job-to-machine assignment, we list schedule the jobs assigned to a particular group on the machines of this group. Precisely, our algorithm is the following.

**Algorithm 7:**
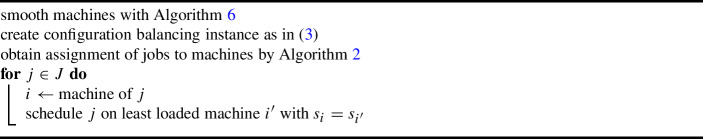
Offline Related Load Balancing

Note that as in the case of unrelated machine load balancing (Theorem 2), we can derandomize this algorithm by employing the GAP LP rounding algorithm by [7] at the loss of another factor 2 in the approximation ratio.

We show that this algorithm gives the desired *O*(1)-approximation. Note that our previous analysis of Theorem 2 gave a $O(\frac{\log m}{\log \log m})$-approximation. We improve on this using the properties of smoothed machines and our adaptive decisions. In the proof, we rely on the following strong bound on the expected maximum of the truncated load; see Appendix A for a proof.

#### Lemma 17

Let $c_1,\ldots , c_m \in \mathbb N_{\ge 1}$ be constants such that $c_i \ge \frac{3}{2} c_{i+1}$ for all $1 \le i \le m$. Let $S_1,\ldots ,S_m$ be sums of independent random variables bounded in $[0,\tau ]$ such that $\mathbb {E}[S_i] \le c_i \tau$ for all $1 \le i \le m$. Then, $\mathbb {E}\big [\max _i \frac{S_i}{c_i}\big ] \le {7}\tau$.

Now we analyze Algorithm 7.

#### Lemma 18

For offline load balancing on related machines with stochastic jobs, Algorithm 7 efficiently outputs an adaptive policy with expected makespan at most $432 \cdot \mathbb {E}[\textsc {Opt}]$.

#### Proof

We let $\textsc {Opt}$ denote the optimal makespan of the input instance and $\textsc {Opt}'$ the optimal makespan after machine smoothing.

The machine smoothing step runs in polynomial time by Lemma 16. As in the case of unrelated machines, the size of the configuration balancing instance is polynomial in the size of the input (since we have *m* configurations per job), so we can solve ($\textsf {LP}_{\textsf {C}}$) in polynomial time. Thus, by Lemma 10, Algorithm 7 runs in polynomial time.

Note that we construct ($\textsf {LP}_{\textsf {C}}$) with respect to the smoothed instance, so our final $\tau$ in Algorithm 2 satisfies $\tau \le 4 \cdot \mathbb {E}[\textsc {Opt}']$ by Lemma 8. Thus, we obtain a (random) job-to-machine assignment with expected truncated load at most $\tau$ on every machine and expected exceptional load at most $\tau$. Summing up the truncated loads within each group with respect to the smoothed instance, we have that the total expected truncated load across all machines in group *k* is at most $m_k \cdot \tau$. Again, we split the makespan of our policy into truncated and exceptional parts

$$ \mathbb {E}\bigg [ \max _{1 \le i \le m} \sum _{j \rightarrow i} X_{ij} \bigg ] \le \mathbb {E}\bigg [ \max _{1 \le i \le m} \sum _{j \rightarrow i} X_{ij}^T \bigg ] + \mathbb {E}\bigg [ \max _{1 \le i \le m} \sum _{j \rightarrow i} X_{ij}^E \bigg ], $$

where the events $j \rightarrow i$ are with respect to our final adaptive assignment. We can upper bound the contribution of the exceptional parts (the latter term) by $\tau$ using (1). It remains to bound the contribution of truncated parts. We do so by considering the makespan on each *group*. For group *k*, we let $j \rightarrow k$ denote the event that we non-adaptively assign job *j* to a machine in group *k*, and $X_{kj}$ be the size of job *j* on any machine in group *k* (recall that they all have the same speed). Then,

$$ \mathbb {E}\bigg [ \max _{1 \le i \le m} \sum _{j \rightarrow i} X_{ij}^T \bigg ] = \mathbb {E}\bigg [\max _k \max _{i \in G_k} \sum _{j \rightarrow i} X_{ij}^T \bigg ], $$

where $G_k$ is the collection of all machines in group *k*. Similar to the analysis of list scheduling by [2], i.e, by an averaging argument, we obtain

$$\begin{aligned} \max _{i \in G_k} \sum _{j \rightarrow i} X_{ij}^T \le \frac{1}{m_k} \sum _{j \rightarrow k} X_{kj}^T + \max _{j \rightarrow k} X_{kj}^T \le \frac{1}{m_k} \sum _{j \rightarrow k} X_{kj}^T + \tau . \end{aligned}$$

Plugging this into our bound on the contribution of truncated parts, we have

$$\begin{aligned} \mathbb {E}\bigg [ \max _{1 \le i \le m} \sum _{j \rightarrow i} X_{ij}^T \bigg ] \le \mathbb {E}\bigg [\max _k \frac{1}{m_k} \sum _{j \rightarrow k} X_{kj}^T \bigg ] + \tau \end{aligned}$$

The expected truncated load on group *k* is at most $m_k \cdot \tau$, and we have $m_k \ge \frac{3}{2} m_{k+1}$ for all *k* by the properties of smoothed instances. Thus, Lemma 17 gives $\mathbb {E}[\max _k \frac{1}{m_k} \sum _{j \rightarrow k} X_{kj}^T] \le 7 \tau$, so we can upper bound the expected contribution of truncated parts by $8 \tau$.

To conclude, combining our bounds on the truncated- and exceptional parts, the expected makespan of our algorithm is at most $9 \tau \le 36 \cdot \mathbb {E}[\textsc {Opt}'] \le 432 \cdot \mathbb {E}[\textsc {Opt}]$, where we use the facts $\tau \le 4 \cdot \mathbb {E}[\textsc {Opt}']$ and $\mathbb {E}[\textsc {Opt}'] \le 12 \cdot \mathbb {E}[\textsc {Opt}]$. $\square$

### Online setting

We apply a similar framework as above. Note that our online configuration balancing algorithm loses a logarithmic factor in the number of resources, so to obtain a $O(\log \log m)$-approximation, we aggregate each group (in the smoothed-machines instance) to a single resource. Intuitively, this definition captures the fact that we will average all jobs assigned to a group over the machines in this group. Thus, our configuration balancing instance will have only $O(\log m)$ resources and applying Theorem 1 proves Theorem 4.

We first describe how to construct the configuration balancing instance. Suppose we have smoothed machines with $m'$ groups. We define $m' + 1$ resources indexed $0, \dots , m'$ such that the 0th resource is a virtual resource collecting exceptional parts, and the resources $1, \dots m'$ index the groups of smoothed machines and collect the respective truncated parts. Each job *j* has $m'$ configurations $c \in [m']$ (one per job-to-group assignment) defined by

4 $$\begin{aligned} X_{kj}(c) = {\left\{ \begin{array}{ll} \mathbb {E}\big [ X_{kj}^E \big ] &{} if k = 0, \\ \frac{1}{m_k} \mathbb {E}\big [ X_{kj}^T\big ] &{} if k = c, \\ 0 &{} otherwise , \end{array}\right. } \end{aligned}$$

for a fixed truncation threshold $\tau$. Note that these configurations are deterministic. Intuitively, this definition captures the fact that we will average all jobs assigned to group *k* over the $m_k$ machines in the group. Now we give our algorithm for the online setting.

**Algorithm 8:**
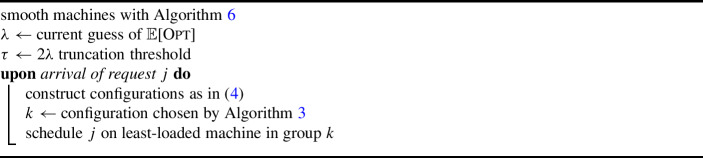
Online Related Load Balancing

#### Lemma 19

For online load balancing on related machines with stochastic jobs, Algorithm 8 runs in polynomial time and correctly solves the subproblem of Lemma 11 for $\alpha = O(\log \log m)$.

#### Proof

It is clear that the algorithm runs in polynomial time.

Let $\textsc {Opt}_{S}$ be the optimal makespan of the smoothed load balancing instance. We first claim that the resulting configuration balancing instance has optimal makespan at most $O(\mathbb {E}[\textsc {Opt}_{S}])$ for any truncation threshold $\tau \ge 2 \mathbb {E}[\textsc {Opt}_{S}]$. To see this, observe that ($\textsf {LP}_{\textsf {C}}$) for the smoothed load balancing instance is feasible for $\tau$. Then, ($\textsf {LP}_{\textsf {C}}$) for the deterministic configuration balancing instance is obtained from this LP by aggregating the truncated constraints for all machines in the same group and dividing by $m_k$. Thus, this latter LP is also feasible for the same threshold, and it admits a constant-factor approximation by Shmoys-Tardos [7]. Thus, $\textsc {Opt}_D$, the optimal solution of the deterministic configuration balancing instance defined by (4), is at most $O(\mathbb {E}[\textsc {Opt}_S])$.

Further, by the properties of smoothed machines, the resulting configuration balancing instance has $m' + 1 = O(\log m)$ resources. As argued in the proof of Theorem 4, the potential function guarantees that Algorithm 3 never creates makespan (for the configuration balancing instance) more than $O(\log \log m) \lambda$ if $\textsc {Opt}_D \le \lambda$. Hence, by (4), the total expected exceptional load and average expected truncated load within each group are at most $O(\log \log m) \lambda$. We translate these bounds into bounds on the makespan for the load balancing instance.

Similar to Lemma 18, we can upper bound the contribution of the exceptional parts by $O(\log \log m) \lambda$ using (1) and the truncated parts by $O(\log \log m) \lambda$ using Lemma 17. Thus, the expected makespan of the load balancing instance is at most $O(\log \log m) \lambda$, as required.

Finally, we recall that if Algorithm 3 fails, then $\textsc {Opt}_D>\lambda$, which implies $\mathbb {E}[\textsc {Opt}_S] \ge \Omega (\lambda )$. $\square$

The proof of Theorem 4 follows immediately from Lemmas 18 and 19.

## Conclusion

We considered the configuration balancing problem under uncertainty. In contrast to the (often overly optimistic) clairvoyant settings and the (often overly pessimistic) non-clairvoyant settings, we consider the stochastic setting where each request *j* presents a set of random vectors, and we need to (adaptively) pick one of these vectors, to minimize the *expected* maximum load over the *m* resources. We give logarithmic bounds for several general settings (which are existentially tight), and a much better *O*(1) offline and $O(\log \log m)$ online bound for the related machines setting. Closing the gap for online related-machines load balancing remains an intriguing open problem. More generally, getting a better understanding of both adaptive and non-adaptive algorithms for stochastic packing and scheduling problems remains an exciting direction for research.

## Appendix A Inequalities and bounds on sums of random variables

In this section, we state some useful probabilistic inequalities. Further, we give the proofs of Lemmas 9, 12, and 17.

### Lemma 20

(Bernstein’s inequality) Let the random variables $X_1, \ldots , X_n$ be independent with $X_i - \mathbb {E}[X_j] \le b$ for each $1\le j \le n$. Let $S=\sum _{j=1}^n X_j$ and let $\sigma ^2 = \sum _{j=1}^n \sigma _j^2$ be the variance of *S*. Then, for any $t >0$,

$$ \mathbb {P}[S > \mathbb {E}[S] + t] \le exp \bigg ( - \frac{t^2}{ 2\sigma ^2 (1 + bt/3\sigma ^2)} \bigg ). $$

### Lemma 21

(Bennett’s inequality [45]) Let $X_1,\dots , X_n$ be independent random variables with zero mean such that $X_j \le a$ for all *j*. Let $S = \sum _j X_j$ and $\sigma ^2 = \sum _j \mathbb {E}[X_j^2]$. Then, for all $t \ge 0$,

$$\begin{aligned} \mathbb {P}(S > t) \le \exp \bigg ( \frac{-\sigma ^2}{a^2} \bigg (\bigg (1 + \frac{at}{\sigma ^2}\bigg )\log \bigg (1 + \frac{at}{\sigma ^2}\bigg ) - \frac{at}{\sigma ^2}\bigg )\bigg ). \end{aligned}$$

### Lemma 22

(Chernoff-Hoeffding type inequality; Theorem 1.1 in [46]) Let $S= \sum _{j=1}^n X_j$ where $X_j$, $1 \le j \le n$, are independently distributed in [0, 1]. If $t > 2e\mathbb {E}[S]$, then

$$ \mathbb {P}[S > t] \le 2^{-t}. $$

Having these inequalities at hand, we are now ready to prove Lemmas 9, 12, and 17, that bound the expected maximum of sums of independent variables.

### Lemma 9

Let $S_1, \dots , S_m$ be sums of independent RVs bounded in $[0,\tau ]$ for some $\tau > 0$ such that $\mathbb {E} [S_i] \le \tau$ for all $i \in [m]$. Then, $\mathbb {E} [\max _i S_i] = O\big (\frac{\log m}{\log \log m}\big )\tau$.

### Proof

By re-scaling, it suffices to prove the lemma for $\tau = 1$. Consider one such sum, say $S = \sum _j X_j$, where the $X_j$s are independent and bounded in [0, 1]. We use Bennett’s inequality (Lemma 21) to bound the upper tail of *S*. Applying Bennett’s inequality to $S - \mathbb {E}[S]$ with $a = 1$ and $\sigma ^2 = \sum _j \mathbb {E}\big [(X_j - \mathbb {E}[X_j])^2\big ] \le \sum _j \mathbb {E}[X_j^2] \le \sum _j \mathbb {E}[X_j] \le 1$ gives for any $t \ge 0$:

$$\begin{aligned} \mathbb {P}[S> 1 + t]&\le \mathbb {P}[S > \mathbb {E}[S]+t]\\&\le \exp \bigg ( - \big (\sigma ^2 + t\big ) \log \bigg (1 + \frac{t}{\sigma ^2}\bigg ) + t \bigg )\\&\le \exp \big ( - t \log (1 + t) + t \big ) = \frac{e^t}{(1 + t)^t}, \end{aligned}$$

where we use $0\le \sigma ^2 \le 1$ in the third inequality. In particular, for $\ell = O\big (\frac{\log m}{\log \log m}\big )$ large enough, we have for any $t \ge 0$:

$$\begin{aligned} \mathbb {P}[S > 1 + \ell + t] \le \frac{e^{\ell + t}}{(1 + \ell + t)^{\ell + t}} \le \frac{e^\ell }{\ell ^\ell } \cdot \frac{e^t}{\ell ^t} = O\bigg (\frac{1}{m}\bigg ) \cdot e^{-t}. \end{aligned}$$

This tail bound holds for all $S_1, \dots , S_m$. Union-bounding over all *m* sums gives:

$$\begin{aligned} \mathbb {E} \big [\max _i S_i\big ]&= \int _0^\infty \mathbb {P}\big [ \max _i S_i> t\big ] \cdot dt\\&\le (1 + \ell ) + \int _0^\infty \mathbb {P}\big [\max _i S_i> 1+ \ell + t\big ] \cdot dt\\&\le (1 + \ell ) + \sum _i \int _0^\infty \mathbb {P}[S_i > 1 + \ell + t] \cdot dt\\&= (1 + \ell ) + m \cdot O\bigg (\frac{1}{m}\bigg ) \int _0^\infty e^{-t} \cdot dt = O(\ell ) = O \bigg (\frac{\log m}{\log \log m} \bigg ). \end{aligned}$$

$$\square $$

### Lemma 12

Let $S_1, \ldots , S_m$ be sums of independent RVs bounded in $[0,\tau ]$ for $\tau >0$ such that $\mathbb {E}[S_i] \le O(\log m) \tau$ for all $1 \le i \le m$. Then, $\mathbb {E}[\max _i S_i] \le O(\log m) \tau$.

### Proof

Let $C \ge 0$ be a constant such that $\mathbb {E}[S_i] \le C \tau \log m$ for all $1 \le i \le m$ and fix $t > 2e C \tau \log m$, where *e* is the Euler constant. Fixing $i\in [m]$, we know that the random variables constituting $S_i$ are independent and bounded by $\tau$. Hence, using the Chernoff-Hoeffding bound in Lemma 22, we have $\mathbb {P}[S_ i > t] < 2^{-t}$. Therefore,

$$\begin{aligned} \mathbb {E}\Big [ \max _{1 \le i \le m} S_i \Big ] \nonumber&= \int _{0}^\infty \mathbb {P}\Big [ \max _{1 \le i \le m} S_i> t \Big ] dt \\&\le O(\log m)\tau + \int _{2eC \tau \log m}^\infty \mathbb {P}\Big [ \max _{1 \le i \le m} S_i> t \Big ] dt \\&\le O(\log m)\tau + \sum _{i=1}^m \int _{2eC \tau \log m}^\infty \mathbb {P}\Big [ S_i > t \Big ] dt \\&\le O(\log m)\tau + m \int _{2 e C \tau \log m}^\infty 2^{-t} dt \\&= O(\log m) \tau , \end{aligned}$$

where the third line uses a union bound over all $i \in [m]$. $\square$

### Lemma 17

Let $c_1,\ldots , c_m \in \mathbb N_{\ge 1}$ be constants such that $c_i \ge \frac{3}{2} c_{i+1}$ for all $1 \le i \le m$. Let $S_1,\ldots ,S_m$ be sums of independent random variables bounded in $[0,\tau ]$ such that $\mathbb {E}[S_i] \le c_i \tau$ for all $1 \le i \le m$. Then, $\mathbb {E}\big [\max _i \frac{S_i}{c_i}\big ] \le {7}\tau$.

### Proof

By re-scaling, we can assume $\tau = 1$. Fix one sum, say $S = \sum _j X_j$ with $\mathbb {E}\big [ \sum _j X_j \big ] \le c$. Towards applying Bernstein’s inequality, we first bound the variance,

$$ Var \bigg [ \frac{S}{c} \bigg ] = \sum _{j} Var \bigg [ \frac{X_j}{c}\bigg ] {\le \sum _j \mathbb {E}\bigg [ \bigg (\frac{X_j}{c}\bigg )^2 \bigg ] \le \frac{1}{c} \sum _j \mathbb {E}\bigg [ \frac{X_j}{c} \bigg ] \le \frac{1}{c}}, $$

where we used the independence of the $X_j$’s, the fact that $\frac{X_j}{c} \le \frac{1}{c}$, and the fact that $\sum _{j } \mathbb {E}[X_j] \le c$. Further, note that $\mathbb {E}\big [\frac{S}{c} \big ] \le 1$ and $\frac{X_j}{c} - \mathbb {E}\big [\frac{X_j}{c}\big ] \le \frac{1}{c}$ for all *j*. Thus, for any $t \ge 1$ Bernstein’s inequality (Lemma 20) guarantees

$$\begin{aligned} \mathbb {P}\bigg [\frac{S}{c} \ge 1 + t\bigg ]&\le \mathbb {P}\bigg [\frac{S}{c} \ge \mathbb {E}\bigg [ \frac{S}{c} \bigg ] + t\bigg ]\\&\le \exp \bigg ( - \frac{t^2}{\frac{2}{c}(1 + t/3)}\bigg )\\&= \exp \bigg ( - c \cdot \frac{t^2}{2 + 2t/3}\bigg )\\&\le \exp \bigg ( - \frac{3c}{8} \cdot t\bigg ) \end{aligned}$$

Union-bounding over all such sums, we obtain for any $t \ge \frac{8}{3}$

$$\begin{aligned} \mathbb {P}\Big [\max _{1 \le i \le m} S_i /c_i \ge 1 + t \Big ]&\le \sum _{i=1}^{m} \exp \bigg ( -\frac{3c_i}{8} t \bigg ) \\&\le \sum _{i=1}^{m} \exp \bigg ( - (3/2)^{m-i} c_{m} \cdot \frac{3t}{8} \bigg ) \\&\le \sum _{i=1}^{m} \exp \bigg ( - (3/2)^{m-i} \cdot \frac{3t}{8} \bigg ) \\&\le \sum _{i=1}^{m} \bigg (\frac{2}{3}\bigg )^{m-i} e^{-3t/8} \\&\le 3 e^{-3t/8}, \end{aligned}$$

where we used $c_i \ge \frac{3}{2} c_{i+1}$ in the second inequality and $c_{m} \ge 1$ in the third inequality. For the fourth inequality, we use that, for $s \ge 1$ and $x \ge 0$, we have $\exp \big ( - s \big (\frac{3}{2}\big )^x \big ) \le \big (\frac{2}{3}\big )^x e^{-s}$. To see this, observe that $s (\frac{3}{2})^x \ge s \big ( x \cdot \log (\frac{3}{2}) + 1 \big )$, using the fact $e^x \ge 1 + x$ for all $x \ge 0$. Since $s \ge 1$, we further have $s (\frac{3}{2})^x \ge x \cdot \log (\frac{3}{2}) + s$. Taking the exponential of both sides and re-arranging gives the desired inequality.

Hence, we conclude the proof with

$$\begin{aligned} \mathbb {E}\big [\max _{1 \le i \le m} S_i/c_i \big ]&\le \big (1 + \frac{8}{3}\big ) + \int _{\frac{8}{3}}^\infty \mathbb {P}\big [ \max _{1 \le i \le m} S_i/c_i > 1 + t \big ] dt \\ {}&\le \frac{11}{3} + 3 \int _{\frac{8}{3}}^\infty e^{-3t/8} dt = \frac{11}{3} + \frac{8}{e} \le 7. \end{aligned}$$

$$\square $$

## Appendix B Clairvoyance gap for load balancing on related machines

In this section, we quantify the power and limitation of non-clairvoyant algorithms for load balancing on related machines. Recall that a non-clairvoyant algorithm has no prior knowledge of the job sizes and must decide where to schedule a job using only the information about current machine loads and speeds. After an algorithm’s decision upon assigning a job, an adversary chooses the realized size of the job, which the algorithm then observes for subsequent decisions. We refer to a non-clairvoyant *online* algorithm as a non-clairvoyant algorithm that learns about the existence of jobs (of unknown size) online one by one.

Our goal is to approximate the optimal makespan achievable by a clairvoyant offline algorithm, which knows all jobs and sizes in advance, by a non-clairvoyant (online) algorithm. We define the *clairvoyance gap* as the maximum ratio between the makespan of an optimal non-clairvoyant algorithm and the optimal makespan achievable by a clairvoyant offline algorithm – precisely $\max _{\mathcal {I}} \frac{\textsc {Opt}_{N\!C}(\mathcal {I})}{\textsc {Opt}_{C}(\mathcal {I})}$, where $\textsc {Opt}_{N\!C}$ and $\textsc {Opt}_{C}$ are the makespan of the optimal non-clairvoyant and clairvoyant algorithms, respectively.

We show the following result.

### Theorem 23

The clairvoyance gap for related machine scheduling is $\Theta (\sqrt{m})$.

To show this result, we first give a lower bound on the clairvoyance gap and then complement it by an upper bound achieved by a non-clairvoyant online list scheduling algorithm.

### Lemma 24

The clairvoyance gap for scheduling on related machines is $\Omega (\sqrt{m})$.

### Proof

Consider an instance with *m* machines, one fast machine with speed 1 and $m-1$ slow machines with speed $\frac{1}{\sqrt{m}}$. There are *m* jobs, one big job with size 1 and $m-1$ small jobs with size $\frac{1}{\sqrt{m}}$. There is a schedule of makespan 1, which is achieved by assigning the big job to the fast machine and one small job per slow machine.

We show that any non-clairvoyant algorithm has makespan $\Omega (\sqrt{m})$ which implies the lemma. To that end, we define the following adversarial strategy: the adversary reveals only small jobs (unless it runs out of small jobs, in which case the final job is big) until the first time that the algorithm decides to use a slow machine. Then the adversary makes this job big.

If an algorithm only uses the fast machine, then it has makespan $1 + (m-1) \cdot \frac{1}{\sqrt{m}} = \Omega (\sqrt{m})$. Otherwise, the algorithm must use a slow machine, which receives the big job from the adversary, which also gives makespan $\sqrt{m}$. Hence, any non-clairvoyant algorithm incurs a makespan $\Omega (\sqrt{m})$. $\square$

In the next lemma, we show an upper bound on the clairvoyance gap. We refer to *list scheduling* as the algorithm that assigns the next job to the currently least-loaded machine. Here, the load of a machine is the total processing time of jobs that have been assigned to the machine. We use list scheduling only on a subset of machines that are “fast enough” and leave the remaining machines idle. More precisely, we list schedule the jobs in the order of their arrival on the machines with speeds within a factor $\frac{1}{\sqrt{m}}$ of the fastest.

### Lemma 25

The clairvoyance gap for related machine scheduling is $O(\sqrt{m})$.

### Proof

For convenience, we re-scale the instance such that the speed of the fastest machine is 1. Hence, our algorithm uses all machines *i* with $s_i \in \big [ \frac{1}{\sqrt{m}},1\big ]$; we call such machines *fast* and the remaining ones *slow*. Clearly, list scheduling on the fast machines is both, online and non-clairvoyant. It remains to bound the competitive ratio. To this end, we observe

$$ C_{\max } \le \frac{\sum _{j \in J} p_j}{\sum _{i: s_i \ge 1/\sqrt{m}} s_i} + \frac{\max _{j \in J} p_j}{\min _{i: s_i \ge 1/\sqrt{m}} s_i}, $$

where the first term bounds the starting time and the second term bounds the processing time of any job. We bound both terms separately by comparing to the optimal solution Opt, where Opt denotes the optimal schedule as well as its makespan. Clearly, $\max _{j \in J} p_j \le \textsc {Opt}$, which implies that the second term is at most $\sqrt{m} \cdot \textsc {Opt}$.

For the first term, note that the total size of jobs that Opt schedules on fast machines is at most $m'\cdot \textsc {Opt}$, where $m'$ is the number of fast machines. The total size of jobs that Opt schedules on slow machines is at most $m \cdot \frac{1}{\sqrt{m}} \cdot \textsc {Opt}= \sqrt{m} \cdot \textsc {Opt}$ as there are at most *m* slow machines. Further, the total speed of the fast machines is at least 1 (because the fastest machine has speed 1) and also at least $m' \frac{1}{\sqrt{m}}$. Therefore,

$$ \frac{\sum _{j \in J} p_j}{\sum _{i: s_i \ge 1/\sqrt{m}} s_i} \le \frac{m' \cdot \textsc {Opt}+ \sqrt{m} \cdot \textsc {Opt}}{\max \{ 1, m'/\sqrt{m} \} } \le \bigg (\frac{m'}{m'/\sqrt{m}} + \frac{\sqrt{m}}{1} \bigg ) \cdot \textsc {Opt}= O(\sqrt{m}) \cdot \textsc {Opt}, $$

concluding the proof. $\square$

## Appendix C Integrality gap of ($\textsf {LP}_{\textsf {C}}$)

In this section, we define and bound the integrality gap of ($\textsf {LP}_{\textsf {C}}$) by $\Omega \big (\frac{\log m}{\log \log m}\big )$; let us recall ($\textsf {LP}_{\textsf {C}}$) here.

For instance $\mathcal I$, let $\tau _{\textsf {LP}}(\mathcal I)$ be the minimal value of $\tau$ such that ($\textsf {LP}_{\textsf {C}}$) is feasible and let $\tau _{\textsf {IP}}(\mathcal I)$ be the minimal value of $\tau$ such that ($\textsf {LP}_{\textsf {C}}$) is feasible with the additional constraint that $y_{cj}$ is integer for every $j \in [n]$ and $c \in [q_j]$ is feasible. Then, *integrality gap* is defined as $\max _{\mathcal {I}} \frac{\tau _{\textsf {IP}}(\mathcal {I})}{\tau _{\textsf {LP}}(\mathcal {I})}$, where the maximum is taken over all instances. Observe that $\tau$ is not only the right-hand side but also the truncation threshold.

To give a lower bound on said integrality gap, it is sufficient to restrict to the special case of congestion minimization and the corresponding linear program ($\textsf {LP}_{\textsf {P}}$). This allows us to use the following theorem from [4], which we rewrite for our purposes. (Note that some of the statement details are actually taken from its proof and not from the original theorem.)

### Theorem 26

(Theorem 3 in [4]) For $k \in \mathbb N$ sufficiently large and any constant $c > 0$, there exists a directed graph $G_k$ with *k* vertices and $m = \Theta (k)$ edges each with unit capacity and a set of unit routing requests $(a_j, b_j, 1)$ for $j \in [n]$ such that there is a fractional flow of congestion at most $O\big (\frac{1}{\log ^c k}\big ) = O\big (\frac{1}{\log ^c m}\big )$ and any integral flow on $G_k$ has a congestion at least $\Omega \big ( \frac{\log k}{\log \log k}\big ) = \Omega \big ( \frac{\log m}{\log \log m}\big )$.

Choosing a truncation threshold $\tau \ge 1$ for the instance guaranteed by Theorem 26 implies that the truncated demand for each request coincides with the actual demand; in particular, the exceptional part equals 0. Hence, on these instances ($\textsf {LP}_{\textsf {P}}$) (and hence ($\textsf {LP}_{\textsf {C}}$)) is equivalent to the following LP, where we use $x_{ej} = \frac{x_j}{s_e} = 1$.

Now we are ready to bound the integrality gap. It is easy to see that a fractional flow on $G_k$ corresponds to a fractional solution of ($\textsf {LP}_{\textsf {P-det}}$): Using the standard flow decomposition, we obtain the amount of flow sent along each path $P \in \mathcal P_j$, and we set $y_{Pj} \le 1$ to be this value. Hence, Theorem 26 guarantees that ($\textsf {LP}_{\textsf {P-det}}$) is feasible for $\tau = 1$. Thus, $\tau _{\textsf {LP}} \le 1$. Conversely, additionally requiring $y_{Pj} \in \{0,1\}$ implies that the (unit) demand of each request needs to be sent along exactly one path; this in turn corresponds to an integral flow in network $G_k$. Hence, Theorem 26 implies that $\tau _{\textsf {IP}} \ge \Omega \big (\frac{\log m}{\log \log m}\big )$ since any integral flow causes congestion at least that big. Combining these two observations indeed implies that the integrality gap of ($\textsf {LP}_{\textsf {C}}$) is at least $\Omega \big (\frac{\log m}{\log \log m}\big )$.

## Appendix D Online guess-and-double

Our online algorithm must find a good guess of $\tau$ such that $\tau = O \big ( \mathbb {E}[\textsc {Opt}] \big )$. This requires dynamically doubling $\tau$ throughout the algorithm once our current guess is too small. This subproblem is captured by the next lemma, re-stated from Sect. 2.

### Lemma 11

Given an instance of online configuration balancing with stochastic requests, suppose there exists an online algorithm that, given parameter $\lambda > 0$, never creates an expected makespan more than $\alpha \cdot \lambda$, possibly terminating before handling all requests. Further, if the algorithm terminates prematurely, then it certifies that $\mathbb {E}[\textsc {Opt}] > \lambda$. Then, there exists an $O(\alpha )$-competitive algorithm for online configuration balancing with stochastic requests. Further, the resulting algorithm preserves non-adaptivity.

The idea again is to guess a good threshold $\tau$, but the main difference is that we must handle requests online on the way, so we need to bound the expected makespan over all doubling steps.

Concretely, let $\mathcal {A}$ be the online algorithm assumed by the lemma. Upon arrival of the first request *j* (before assigning it), we initialize $\tau = \min _c \, \mathbb {E}[\max _i X_{ij}(c)] \le \mathbb {E}[\textsc {Opt}]$. Note we may assume the first request is non-trivial, i.e. not every configuration is the zero vector, so our initial guess satisfies $\tau > 0$. We begin running $\mathcal {A}$ with parameter $\lambda = \tau$. If $\mathcal {A}$ terminates before handling all requests, then we double $\tau$ and re-run $\mathcal {A}$ with the doubled guess on the remaining requests.

As before, we need to show our final guess satisfies $\tau = O \big ( \mathbb {E}[\textsc {Opt}] \big )$. This holds if the algorithms handles all requests for the initial guess. Otherwise, in every run of $\mathcal {A}$ except the final one, we certify $\mathbb {E}[\textsc {Opt}] > \tau$, so our final guess of $\tau$ satisfies $\tau \le 2 \cdot \mathbb {E}[\textsc {Opt}]$. Note that the expected makespan of this run is at most $\alpha \cdot \lambda = O(\alpha ) \cdot \mathbb {E}[\textsc {Opt}]$.

The total expected makespan over all runs is upper bounded by the sum of expected makespans of each run. We can upper bound the expected makespan of a run by $\alpha$ times the $\tau$ guess for that run, which geometrically decreases by a 2-factor in each previous run. We conclude, the total expected makespan is also at most $O(\alpha ) \cdot \mathbb {E}[\textsc {Opt}]$, as required.
